# Fn14 is an activity-dependent, Bmal1-regulated cytokine receptor that induces rod-like microglia and restricts neuronal activity *in vivo*

**DOI:** 10.1016/j.celrep.2026.116926

**Published:** 2026-01-29

**Authors:** Austin Ferro, Dominic J. Vita, Trevor Fallon, Anosha Arshad, Leah Boyd, Tess Stanley, Qianyu Lin, Adrian Berisha, Uma Vrudhula, Adrian M. Gomez, Irene Sanchez-Martin, Jeremy C. Borniger, Lucas Cheadle

**Affiliations:** 1 Cold Spring Harbor Laboratory, Cold Spring Harbor, NY 11740, USA; 2 Department of Neurobiology and Behavior, Stony Brook University Renaissance School of Medicine, Stony Brook, NY 11794, USA; 3 Howard Hughes Medical Institute, Cold Spring Harbor, NY 11740, USA; 4 These authors contributed equally; 5 Lead contact

## Abstract

Cytokines and their receptors play important roles in brain development and aging-related disease, but their functions within the healthy adult brain remain poorly understood. Here, we show that pyramidal neurons in hippocampal CA1 induce Fn14 expression in response to activity and environmental enrichment. Once expressed, Fn14 dampens the activity of these neurons most prominently at the daily transition between light and dark. Fn14 expression in CA1 neurons is regulated by the circadian transcription factor Bmal1, and mice lacking Fn14 exhibit disrupted sleep-wake patterns *in vivo*. At the cellular level, microglia contact fewer excitatory synapses in the absence of Fn14, while neuronal overexpression of Fn14 induces rod-like microglia and recruits them to excitatory synapses. Beyond a homeostatic context, mice lacking Fn14 exhibit heightened susceptibility to chemically induced seizures. These findings reveal that pro-inflammatory cytokine receptors such as Fn14 can play major roles in healthy neurological function in the adult brain.

## In brief

Ferro et al. show that *Fn14* is induced by environmental enrichment in neurons of CA1, where it recruits rod-like microglia to synapses and dampens neuronal activity. *Fn14* expression is regulated by the circadian transcription factor Bmal1, consistent with circadian cycle length and sleep-wake states being altered in mice lacking Fn14.

## Graphical abstract

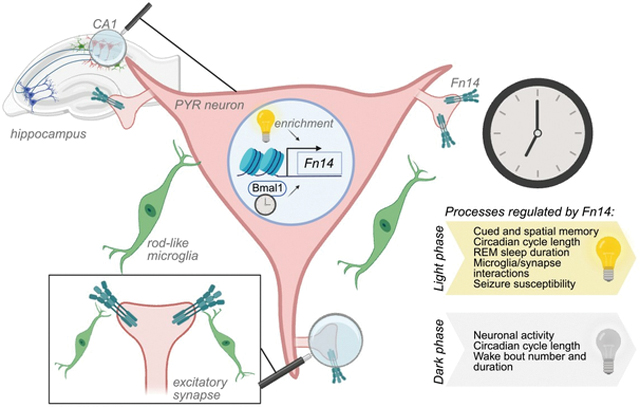

## INTRODUCTION

Despite the long-held view of the nervous system as an immunologically privileged site, cytokines and their receptors are integral to brain development.^1^ For example, cytokine signaling between the brain’s resident immune cells, microglia, and neurons contributes to the assembly and/or elimination of synapses in early life.^2–8^ At the other end of the lifespan, immune signaling molecules can become inappropriately activated during aging, contributing to conditions such as Alzheimer’s disease (AD).^9–11^ However, while cytokines are understood to influence the developing and aging brain, how they mediate adult brain function in the absence of disease remains largely unexplored.

Several observations suggest that cytokine pathways may be uniquely poised to regulate adult brain function. For example, cytokines represent a promising mechanism to mediate interactions between brain cells that are not in direct contact with one another.^12,13^ In addition, just as synapses in the developing brain undergo dynamic changes in structure and physiology, synapses are similarly remodeled in the mature brain, potentially through shared cytokine-driven mechanisms.^14,15^ Finally, there are likely to be evolutionarily conserved benefits of the immune and nervous systems sharing a molecular language in the form of cytokine pathways, such as the facilitation of brain-body interactions.^16,17^ Thus, interrogating the ways in which cytokines and cytokine receptors operate within the adult brain may illuminate new modes of neuroimmune communication within the brain and beyond.

The TWEAK-Fn14 pathway has emerged as a promising candidate to mediate adult brain function. In this pathway, the tumor necrosis factor (TNF) family cytokine TWEAK (TNF-associated weak inducer of apoptosis) binds to the TNF receptor (TNFR) family member Fn14 (fibroblast-growth-factor-inducible protein 14 kDa), thereby eliciting local cellular remodeling events alongside changes in gene expression that underlie processes such as inflammation, tissue regeneration, and angiogenesis.^18–22^ We recently identified a requirement of TWEAK-Fn14 signaling between microglia and excitatory neurons for the refinement of visual circuit connectivity between the retina and the dorsal lateral geniculate nucleus (dLGN) of the thalamus, unveiling a key role for Fn14 in neural circuit development.^5,23,24^ However, whether and how Fn14 mediates mature brain function was not known.

In this study, we harness the cytokine receptor Fn14 as a molecular handle to shed light on the roles of cytokine signaling in the mature brain. We find that Fn14 expression is upregulated in a subset of glutamatergic pyramidal (PYR) neurons in the CA1 subregion of the hippocampus in response to neuronal activity. Subsequently, Fn14 dampens the activity of these neurons in a time-of-day-dependent manner, consistent with molecular and behavioral evidence that Fn14 regulates aspects of hippocampal and circadian function. Mechanistically, we show that Fn14 induces rod-like microglia and promotes interactions between microglia and excitatory synapses. Thus, Fn14 is not only instrumental for circuit development; it mediates features of circuit remodeling and function in the adult brain as well.

## RESULTS

### Excitatory neurons are the predominant expressers of Fn14 in the adult mouse brain

**Figure 1.**
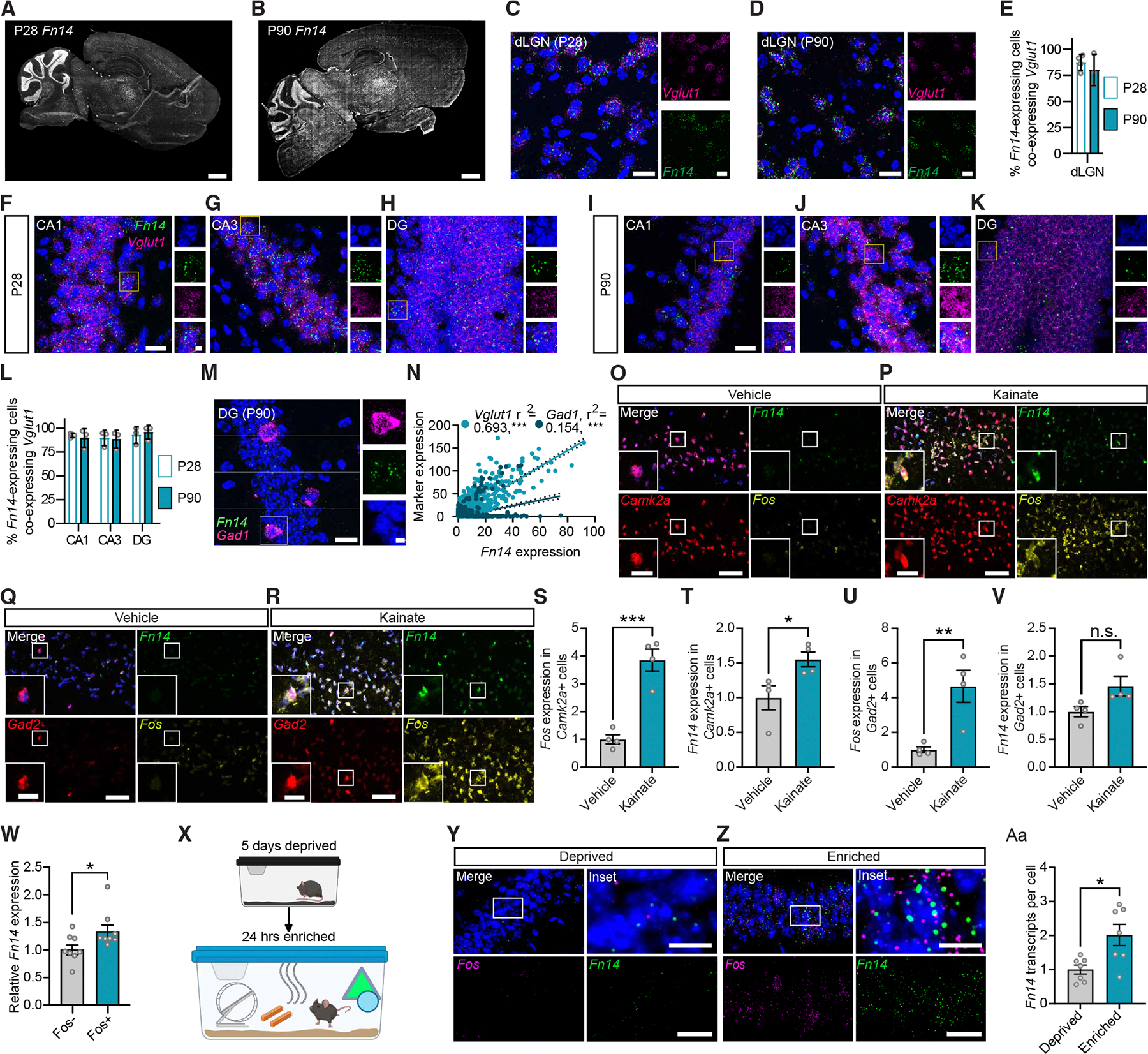
Neuronal activity and environmental enrichment induce *Fn14* expression in pyramidal neurons of hippocampal CA1 (A and B) Confocal images of sagittal sections of the mouse brain at P28 (A) and P90 (B) subjected to single-molecule fluorescence *in situ* hybridization (smFISH) to label *Fn14* mRNA (white). Scale bars, 1 mm. (C and D) High-resolution confocal images of the dLGN in coronal sections from a P28 (C) and a P90 (D) mouse brain probed for *Fn14* (green) and the glutamatergic neuronal marker *Vglut1* (magenta). DAPI is shown in blue. Scale bars, 20 μm. (E) Quantification of the percentage of *Fn14*-expressing cells that also express *Vglut1* in the dLGN at P28 and P90. Unpaired Student’s *t* test, *p* > 0.05; *n* = 3 mice/age. (F–I) Confocal images of CA1 (F), CA3 (G), and dentate gyrus (DG; H) subregions of the hippocampus in a coronal section from a P28 mouse brain probed for *Fn14* (green) and *Vglut1* (magenta). DAPI is shown in blue. Scale bars, 20 μm. Inset scale bar, 5 μm. (I–K) Confocal images of CA1 (I), CA3 (J), and DG (K) regions of the hippocampus in a coronal section from a P90 mouse brain probed for *Fn14* (green) and *Vglut1* (magenta). DAPI is shown in blue. Scale bar, 20 μm. Inset scale bar, 5 μm. (L) Quantification of *Fn14*-expressing cells that are positive for *Vglut1* in the hippocampus at both ages. Two-way ANOVA: region, *p* > 0.05; age, *p* > 0.05; and interaction, *p* > 0.05; *n* = 3 mice/age for each region. (M) Confocal image of *Fn14* (green) and the inhibitory neuron marker *Gad1* (magenta) in the DG at P90. Scale bar, 20 μm. Inset scale bar, 5 μm. (N) Scatterplot demonstrating the correlation between *Fn14* expression (*x* axis) and the expression of excitatory (*Vglut1*) or inhibitory (*Gad1*) neuron markers (*y* axis) in the hippocampus. Linear regression with slope comparison, ***p < 0.001. (O and P) Confocal images of CA1 following smFISH for *Fn14* (green), the pyramidal (PYR) neuron marker *Camk2a* (red), and the activity-dependent gene *Fos* (yellow) in mice exposed to vehicle (O) or kainate (P). Scale bars, 50 μm. Inset scale bars, 16 μm. (Q and R) Confocal images of CA1 following smFISH for *Fn14* (green), the interneuron marker *Gad2* (red), and the activity-dependent gene *Fos* (yellow) in mice exposed to vehicle (Q) or kainate (R). Scale bars, 50 μm. Inset scale bars, 16 μm. (S and T) Quantification of *Fos* (S) or *Fn14* (T) expression in *Camk2a*^+^ neurons in response to vehicle or kainate exposure; values are normalized to vehicle. (U and V) Quantification of *Fos* (U) or *Fn14* (V) expression in *Gad2*^+^ interneurons in response to vehicle or kainate exposure; values are normalized to vehicle. Statistics for (S)–(V): unpaired Student’s *t* test, ***p < 0.001, **p < 0.01, and *p < 0.05; n.s., *p* > 0.05; *n* = 4 mice/condition. (W) Quantification of *Fn14* expression in *Fos*^−^ and *Fos*^+^ PYR neurons aggregated from both vehicle and kainate conditions. Unpaired Student’s *t* test, *p < 0.05. (X) Schematic of the environmental enrichment paradigm depicting 5 days of deprived housing followed by 24 h of enrichment. (Y and Z) Confocal images of CA1 following smFISH for *Fn14* (green) and *Fos* (magenta) in deprived (Y) or enriched conditions (Z). Inset shown in the upper right. Scale bars, 50 μm. Inset scale bars, 10 μm. (AA) Quantification of *Fn14* transcripts per cell in deprived and enriched conditions, normalized to deprived. Unpaired Student’s *t* test, *p < 0.05; *n* = 7 mice/condition. See also Figure S1.

To characterize the roles of Fn14 beyond development, we first asked whether Fn14 is expressed in the adult brain. Toward this end, we quantified *Fn14* mRNA expression in sagittal sections of the mouse brain at postnatal day (P) 28, when brain maturation is nearing completion, and in the fully mature brain at P90 using single-molecule fluorescence *in situ* hybridization (smFISH, RNAscope). At both ages, we observed *Fn14* expression in a subset of cells across a diversity of brain structures. *Fn14* expression generally increased along an anterior-to-posterior axis and was particularly high in the cerebellum, where it was largely restricted to the granule cell layer. *Fn14* was also observed in the brain stem, the dLGN and other thalamic nuclei, and select cells in the hippocampus and cortex (Figures 1A and 1B).

We next assessed the colocalization of *Fn14* with the excitatory glutamatergic neuron marker *Vglut1* and the inhibitory neuron marker *Gad1* in two brain regions: the dLGN and the hippocampus. In the dLGN, a region in which *Fn14* expression is relatively high, as previously reported,^23^ the majority of *Fn14*^+^ cells (~90%) at both P28 and P90 also expressed *Vglut1*, indicating that Fn14 is most highly expressed in excitatory neurons in this region (Figures 1C–1E). Next, we more closely examined *Fn14* expression in the hippocampus for the following reasons: (1) the hippocampus is essential for a plethora of critical brain functions that require synaptic plasticity, most notably learning and memory; (2) hippocampal organization and connectivity have been well characterized; and (3) numerous physiological and behavioral paradigms have been developed to interrogate hippocampal function. Quantification of *Fn14* expression in the three main interconnected hippocampal subregions (the dentate gyrus [DG], CA1, and CA3) at P28 and P90 revealed that, as in the dLGN, about 90% of *Fn14*^+^ cells also expressed the excitatory neuron marker *Vglut1*, which in the hippocampus labels PYR neurons (Figures 1F–1L). Although *Fn14* expression was most frequently observed in excitatory neurons, *Fn14* was expressed in a subset of *Gad1*^+^ inhibitory neurons as well (Figure 1M). On a cell-by-cell basis, the inhibitory neurons that expressed *Fn14* expressed it at a greater level than excitatory neurons, by about 30% (Figure S1A). Consistent with these observations, *Fn14* expression in the hippocampus was positively correlated with the expression of both *Vglut1* (r^2^ = 0.693; *p* < 0.001) and *Gad1* (r^2^ = 0.154; *p* < 0.001; Figure 1N). Conversely, we did not detect appreciable levels of *Fn14* in microglia, oligodendrocyte precursor cells (OPCs), oligodendrocytes, or astrocytes within CA1 (Figures S1B–S1I). Together, these results demonstrate that the majority of *Fn14* transcripts within the hippocampus are localized to excitatory PYR neurons, with some expression in inhibitory neurons as well.

### Neuronal activity induces Fn14 expression in a subset of PYR neurons in hippocampal CA1

Memory encoding and retrieval are core functions of the hippocampus and occur, in part, through the coordination of activity-dependent gene programs that are induced in neurons downstream of synaptic activity. To test the hypothesis that *Fn14* may represent one such inducible gene, we performed smFISH on the CA1 regions of the hippocampi of mice that had been systemically exposed to kainate (10 mg/kg intraperitoneally or water as vehicle control) for 2 h. Kainate is a soluble compound that can cross the blood-brain barrier and bind a subset of glutamate receptors to induce the robust activation of neurons. In hippocampal slices from kainate- or vehicle-treated mice, we probed for *Fn14* along with the excitatory PYR neuron marker *Camk2a*, the inhibitory neuron marker *Gad2*, and *Fos*, an activity-regulated gene that served as a positive control.^25^ As expected, *Fos* was significantly upregulated in both PYR and inhibitory neurons in CA1 following kainate exposure, validating kainate as a robust driver of neuronal-activity-dependent transcription *in vivo* (Figures 1O–1S and 1U).

Similar to *Fos*, *Fn14* expression was also significantly higher in *Camk2a*^+^ excitatory neurons in kainate-treated mice than in vehicle-treated controls (Figures 1O, 1P, and 1T). Conversely, *Fn14* expression within *Gad2*^+^ inhibitory neurons was not significantly altered by neuronal activation, although a trend toward increased expression was observed (Figures 1Q, 1R, and 1V). Consistent with these findings, western blot analysis revealed a significant increase in Fn14 protein in hippocampal lysates from kainate-exposed compared to control mice (Figures S1J–S1L).

Two possible scenarios could give rise to the increase in *Fn14* expression observed in CA1 following kainate exposure: (1) the number of PYR neurons expressing *Fn14* could increase or (2) the number of PYR neurons expressing *Fn14* may remain the same, but these neurons may express a greater amount of *Fn14* when activity is heightened. Our data revealed that the number of PYR neurons expressing *Fn14* was not altered by kainate exposure, supporting the latter interpretation that a subset of PYR neurons expresses *Fn14* more highly in response to activity (Figure S1M), which was true for inhibitory neurons as well (Figure S1N). While kainate is a powerful stimulant that can activate neurons to an extent that is greater than what typically occurs *in vivo*,^26^ we found that Fn14 expression was significantly higher in PYR neurons that expressed *Fos* (i.e., neurons that were recently activated) than in neurons that were *Fos* negative, regardless of whether a mouse was exposed to kainate or vehicle (Figure 1W). These observations are consistent with a scenario in which *Fn14* is transcribed in a distinct cohort of activated PYR neurons at a given time.

To validate these results, we assessed two whole-transcriptome datasets describing the transcriptional responses of hippocampal neurons to kainate *in vivo*.^27,28^ In both datasets, *Fn14* was identified as being significantly induced by neuronal activation, confirming our findings (Figures S1O–S1Q). Interestingly, among the TNFR superfamily members included in the study from Pollina et al.,^27^ 9 of the 18 genes encoding TNFRs exhibited either a significant upregulation (6 genes) or downregulation (3 genes) following kainate exposure compared to vehicle-treated controls (Figure S1O). Thus, TNFRs other than Fn14 may also play important roles in the mature brain that have yet to be dissected. That said, among the six TNFRs that were upregulated by activity in the dataset, *Fn14* was by far the most strongly induced, underscoring that the functions of Fn14 in the brain are not likely to be redundant with the roles of other TNFRs. Overall, these findings reveal that Fn14 is not only most prominently expressed within excitatory PYR neurons among all cell types in CA1; it is also upregulated by neuronal activity within this population.

### Environmental enrichment induces Fn14 expression in CA1 neurons

Since kainate is a strong pharmacological stimulant that can increase neuronal activity to non-physiological levels, we next asked whether CA1 neurons also induced *Fn14* in response to stimuli that are more physiologically relevant to the hippocampus. To this end, we adopted an environmental enrichment paradigm similar to those commonly used to assess hippocampal responses to features of the external environment.^28^ In this paradigm, mice were singly housed in conventional cages with normal bedding in the dark for 5 days and then placed in a much larger cage filled with enrichment devices (e.g., running wheels, huts, tunnels, hanging beads, etc.) under constant light conditions for 24 h (Figure 1X). Exposure of mice to this novel environment doubled *Fn14* expression in CA1 neurons, demonstrating that physiological cues can induce neuronal levels of *Fn14* similarly to kainate (Figures 1Y–1AA). Conversely, even under environmental enrichment conditions, we still detected very low levels of *Fn14* within microglia, OPCs, oligodendrocytes, and astrocytes (Figures S1B–S1I). Altogether, these results suggest that Fn14 may be poised to mediate the functions of hippocampal CA1 in adult mice.

### Fn14 is dispensable for learning but required for cued and spatial memory

**Figure 2.**
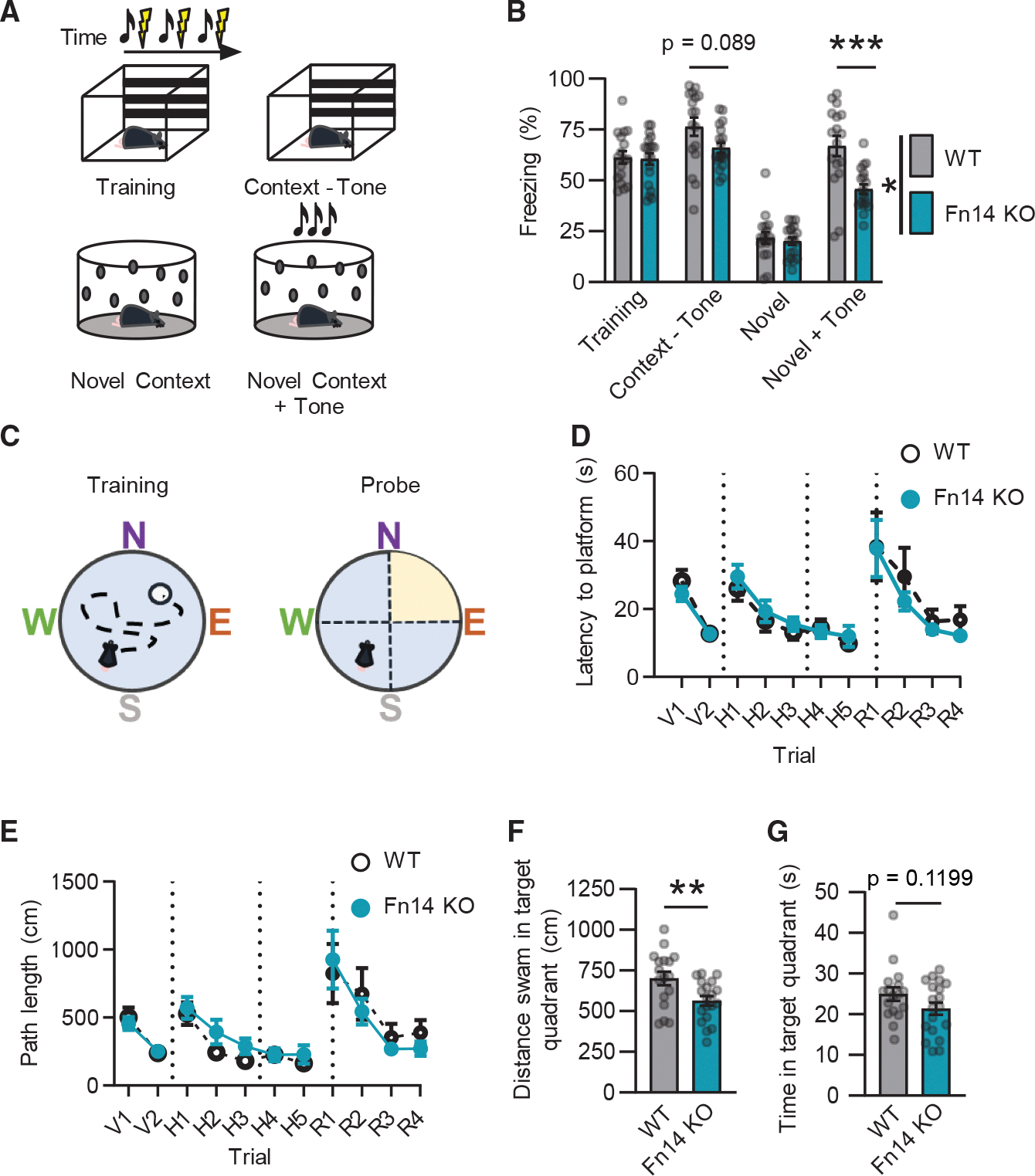
Fn14 is dispensable for learning but required for cued and spatial memory (A) Diagram of the cued fear conditioning (CFC) paradigm. An auditory tone and a unique spatial context were initially paired with an aversive foot shock. The ability of mice to remember this association was later tested by exposing the mice to either the spatial context or the auditory tone in the absence of the shock. Freezing behavior, which mice exhibit when afraid, serves as a readout for how well the mice remember the association between conditioned and unconditioned stimuli. (B) Quantification of percentage of time spent freezing across all conditions. Repeated measures ANOVA: trial, *p* < 0.0001; genotype, *p* < 0.05; subject, trial × genotype, *p* < 0.0001. Bonferroni-corrected multiple comparisons, WT vs. KO: training, *p* > 0.05; context − tone, *p* = 0.089; novel context, *p* > 0.05; novel context + tone, *p* < 0.001. (C) Diagram of Morris water maze (MWM) training and probe trials. (D) Latency to goal platform swum during MWM trials. Repeated measures ANOVA with Šídák’s multiple comparisons test: platform is visible (V), genotype, *p* > 0.05, trial, *p* < 0.0001, trial × genotype, *p* > 0.05; platform is hidden (H), genotype, *p* > 0.05, trial, *p* < 0.0001, trial × genotype, *p* > 0.05; platform goal zone is reversed (R), genotype, *p* > 0.05, trial, *p* < 0.001, trial × genotype, *p* > 0.05. (E) Path length swum by Fn14 WT and KO mice during the MWM test. Repeated measures ANOVA with Šídák’s multiple comparisons test: V, genotype, *p* > 0.05, trial, *p* < 0.0001, trial × genotype, *p* > 0.05; H, genotype, *p* > 0.05, trial, *p* < 0.0001, trial × genotype, *p* > 0.05; R, genotype, *p* > 0.05, trial, *p* < 0.001, trial × genotype, *p* > 0.05. Same *n* as in (B). (F) Distance swum by mice in the target quadrant during the probe trial (cm). Unpaired Student’s *t* test, **p < 0.01. (G) Time spent in target quadrant during probe trial (seconds). Unpaired Student’s *t* test, *p* = 0.12. **p < 0.01 and ***p < 0.001. For all quantifications, *n* = 17 WT and 19 Fn14-KO mice. See also Figure S2.

Hippocampal CA1 is a crucial facilitator of learning and memory. Thus, we next asked whether learning and/or memory was disrupted in the absence of Fn14. To this end, we analyzed learning and memory in Fn14-knockout (KO) mice^23,29^ alongside wild-type (WT) littermates using two behavioral paradigms: cued fear conditioning (CFC) and Morris water maze (MWM). In the CFC task, we examined the abilities of Fn14-KO and WT mice to associate both an auditory (i.e., sensory) cue and a defined spatial context with a paired aversive foot shock (Figure 2A). During the initial conditioning phase, when the foot shock was accompanied by an audible tone (75 dB; 2,000 Hz) and a novel arena (striped walls and floor grating), both Fn14-KO and WT mice exhibited a stereotyped freezing response reflecting fear of the shock. Similarly, when mice of both genotypes were placed into a novel, unfamiliar context (a round arena with polka dotted walls) without a tone, they exhibited low levels of freezing. Next, the mice were subjected to probe trials in which they were exposed to (1) the shock-associated spatial context or (2) the shock-associated auditory tone in the absence of an accompanying foot shock. Fn14-KO mice froze less frequently than WTs when re-exposed to the spatial context (although this trend did not reach statistical significance), and they exhibited significantly less freezing when re-exposed to the auditory tone while in a novel environment (45.8 vs. 66.9 s; Figure 2B). This deficit could reflect an inability of mice lacking Fn14 to generalize their association of the tone with the foot shock to a new spatial context. Together, these data indicate that Fn14 likely contributes to the encoding and/or retrieval of memories, with the strongest deficits in Fn14-KO mice revolving around an inability to pair a sensory cue with an aversive stimulus.

Because impairments in the CFC task could reflect functional changes in the amygdala or the frontal cortex in addition to the hippocampus, we next examined whether the loss of Fn14 would have a similar effect on a more purely hippocampal-dependent spatial learning task, the MWM. In this task, the mice were placed in a round pool with each cardinal direction marked by a distinctive shape and color to allow for spatial mapping of the arena (Figure 2C). During the initial training stage, WT and Fn14-KO mice were both able to effectively locate a visible goal platform. After mice were trained to perform the task, the water in the pool was made opaque and the goal platform submerged, promoting the use of spatial orientation-based strategies for locating the goal platform, rather than the platform itself.^30^ In all trials in which the platform was hidden, WT and Fn14-KO mice learned to find the platform equally well as revealed by their similar latencies to reach the platform and the lengths of the paths that they took to reach it (Figures 2D and 2E). Thus, as also demonstrated by the results of the CFC task, loss of Fn14 does not have a strong observable effect on learning.

To assess spatial memory function, we next tested whether, after a period of 24 h, the mice remembered the location of the hidden platform. When the platform was removed from the pool in probe trials, WT mice swam a significantly greater distance in the quadrant where the platform was previously hidden than Fn14-KO mice by about 25%, suggesting that WT mice were able to remember the location of the platform while mice lacking Fn14 did so less effectively (Figure 2F). As expected, the decreased distance swum in the goal quadrant by the Fn14-KO mice corresponded to a trend toward less time spent in the target quadrant (Figure 2G). These deficits were not caused by an impairment in visual or motor function, as WT and Fn14-KO mice swam an equal distance overall during the probe trial, and Fn14-KO mice exhibited normal visual acuity as assessed by optomotor testing (Figure S2). Following the probe trials, the goal platform was re-introduced into the pool, but now in the opposite quadrant of the arena. Just as in the hidden trials, both WT and Fn14-KO mice were able to learn the new reversed goal zone equally well, again suggesting that Fn14 does not affect the acquisition of new information (Figures 2D and 2E). Thus, Fn14 is dispensable for learning but required for memory, a neurological feature that is controlled by hippocampal CA1.

### Fn14 dampens PYR neuron activity in a time-of-day-dependent manner

The observation that PYR neurons in hippocampal CA1 induce *Fn14* expression in response to environmental enrichment, alongside the discovery that Fn14-KO mice exhibit memory deficits compared to WT mice, led us to next examine the impact of Fn14 loss of function on the activity states and physiological properties of CA1 PYR neurons. To address this question *in vivo*, we performed fiber photometry in awake, behaving Fn14-KO and WT mice. Briefly, this approach employs the viral transduction of excitatory PYR neurons in CA1 with the genetically encoded calcium indicator GCaMP6f downstream of the Camk2a promoter, allowing for specific transduction of excitatory CA1 PYR neurons.^31^ Following viral infection, an optic fiber is implanted over CA1 to detect changes in the amount of internal Ca^2+^ via changes in GCaMP6f fluorescence (ΔF/F), which serves as a surrogate readout of aggregate neuronal activity. Ca^2+^ transients or events, defined as temporal loci at which changes in ΔF/F meet a minimum threshold, are then quantified as a reflection of the activity states of the cells being recorded.

**Figure 3.**
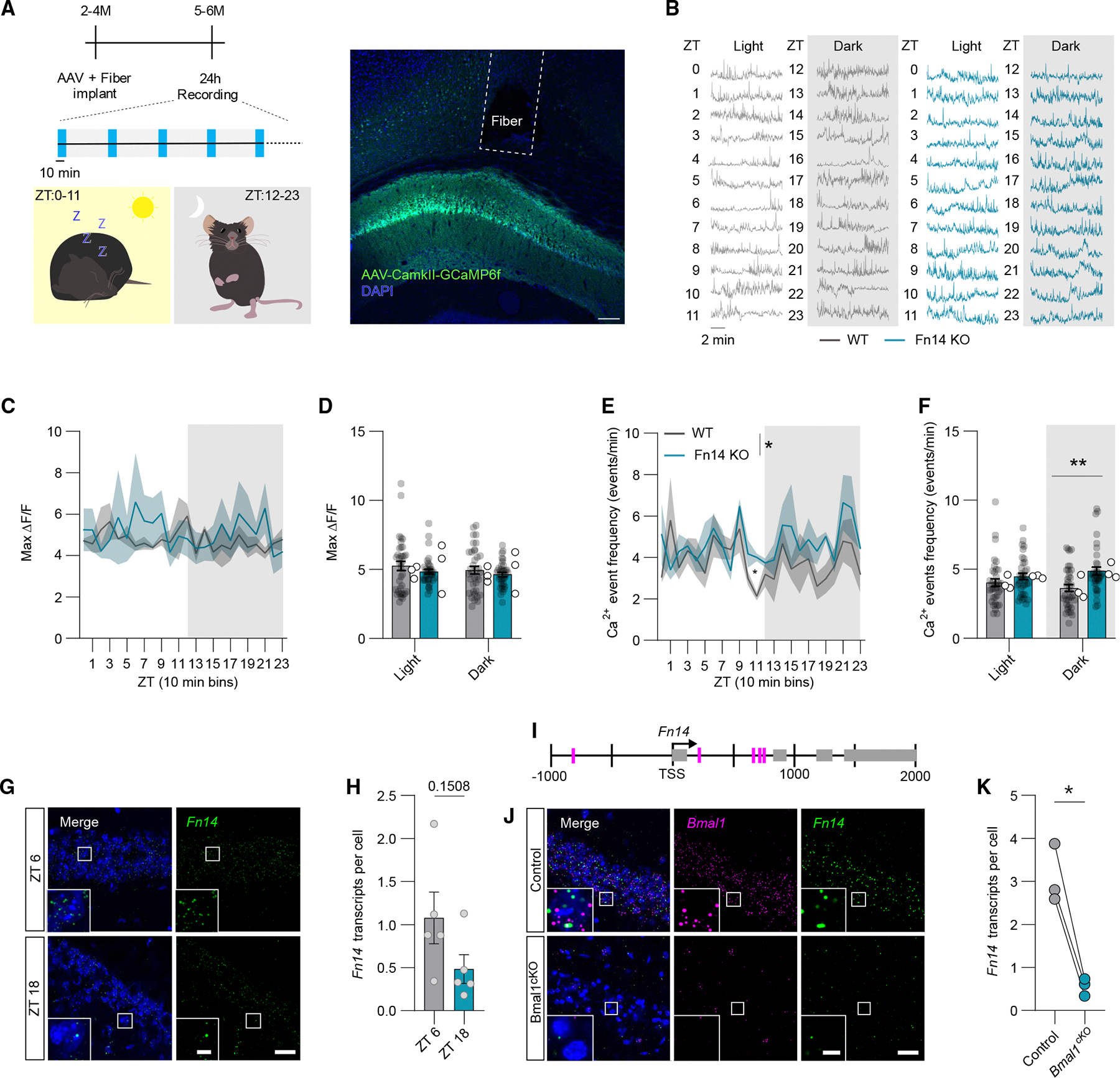
Fn14 is a Bmal1 target gene that dampens neuronal activity in a time-of-day-dependent manner (A) Schematic of the experimental timeline with an example confocal image of GCaMP6f expression in CA1 and the optic fiber tract right above CA1. Scale bar, 100 μm. ZT, zeitgeber time (mouse’s subjective time of day). Blue bars, 10-min recording periods. M, months of age. H, hours. (B) Example 10-min binned calcium traces (ΔF/F) from a representative WT and Fn14-KO mouse, recorded every hour (ZT 0–23) over a single day. (C) Maximum ΔF/F signal over each time bin. Repeated measures two-way ANOVA: time, *p* > 0.05; genotype, *p* > 0.05; interaction, *p* > 0.05. (D) Analysis of maximum ΔF/F signal during the light and dark phases of the day plotted as *Z* scores. Repeated measures two-way ANOVA: time, *p* > 0.05; genotype, *p* > 0.05; interaction, *p* > 0.05. (E) Ca^2+^ event frequency in WT and Fn14-KO mice over a 24-h recording period. Repeated measures two-way ANOVA: time, *p* > 0.05; genotype, *p* < 0.05; interaction, *p* > 0.05; with Tukey *post hoc* test: *p < 0.05 at ZT 11. (F) Quantification of the Ca^2+^ event frequency during the light (ZT 0–11) and dark (ZT 12–23) phases of the day. Repeated measures ANOVA: time, *p* > 0.05; genotype, *p* < 0.01; interaction, *p* > 0.05; with Tukey *post hoc* test: **p < 0.01 during the dark phase. For all fiber analyses, *n* = 36 traces from 3 mice per genotype. Line graphs and histograms show mean ± SEM, while histograms show both acquisitions (closed circles) and within-mouse averages (open circles). (G) Confocal images of smFISH in hippocampal CA1 probing against *Fn14* (green) at ZT 6 (1:00 p.m., top) and ZT 18 (1:00 a.m., bottom). DAPI is in blue. Scale bar, 50 μm. Inset scale bar, 10 μm. (H) Quantification of *Fn14* transcripts per cell in CA1 at ZT 6 and ZT 18 normalized to ZT 6. Unpaired Student’s *t* test, *p* = 0.1508; *n* = 5 mice/time point. Data points represent individual mice with mean ± SEM shown. (I) Schematic depicting predicted Bmal1 binding sites (magenta) within ±1,000 bp of the *Fn14* transcription start site (TSS). Black arrow depicts the start of the *Fn14* gene, gray rectangles represent *Fn14* exons, black lines indicate 500-bp segments relative to the *Fn14* TSS. (J) Confocal images of smFISH in hippocampal CA1 of Bmal1 conditional knockout (Bmal1^cKO^) mice, probing against *Bmal1* (magenta) and *Fn14* (green). Control hemisphere (top), CA1 neurons were transduced with eBFP2. Bmal1^cKO^ hemisphere (bottom), CA1 neurons were transduced with Cre-GFP to elicit ablation of Bmal1 (see related Figures S3C and S3D for validations). DAPI is shown in blue. Scale bar, 50 μm. Inset scale bar, 10 μm. (K) Quantification of *Fn14* transcripts per cell in control and Bmal1^cKO^ hemispheres. Paired *t* test, *p < 0.05. Data points represent individual mice, *n* = 3 mice, 3 hemispheres/condition. See also Figure S3.

To determine whether Fn14 influences the activity of PYR neurons *in vivo*, we assessed the maximum amplitude, as well as the frequency, of Ca^2+^ transients in CA1 PYR neurons from Fn14-KO and WT littermates over a 24-h period during normal home-cage behavior (Figures 3A and 3B). While we did not observe differences in the maximum amplitude (ΔF/F) of Ca^2+^ events between genotypes (Figures 3C and 3D), Fn14-KO mice exhibited a higher frequency of Ca^2+^ transients than WT controls (Figures 3E and 3F). This result suggests that PYR neurons (or, most likely, a subset thereof) are more active in Fn14-KO mice than in WT littermates. Next, we assessed how differences in PYR neuron activity between WT and Fn14-KO mice fluctuated across 24 h. Strikingly, we found that the extent to which the loss of Fn14 increased Ca^2+^ transient frequency varied substantially by time of day. For example, CA1 PYR neurons in the KO demonstrated the most robust increase in Ca^2+^ event frequency over PYR neurons in WT mice at zeitgeber time (ZT) 11, an hour before lights are turned off in the mouse facility and mice transition from less active to more active states (Figures 3A–3E). Furthermore, when we isolated and aggregated the frequency of Ca^2+^ events exhibited during the light and dark periods (light, ZT 0–11; dark, ZT 12–23), we found that Fn14-KO mice exhibited an increase in Ca^2+^ transient frequency only during the dark phase, when mice are more active (Figure 3F). These data suggest that Fn14 constrains the activity of PYR neurons under normal physiological conditions in a time-of-day-dependent manner.

We next sought to corroborate the finding that neurons lacking Fn14 are more active than their WT counterparts at the molecular level by assessing the expression and activation of the activity-dependent transcription factors Fos and Jun in whole-brain homogenates from Fn14-KO and WT mice using ELISAs. Fos and Jun are members of the AP1 family of transcription factors that are activated by neuronal excitation, and they are also targets of the MAPK and JNK/p38 pathways, which can be directly regulated by Fn14.^19,32^ Consistent with neurons being overly active in the absence of Fn14, we observed significantly increased levels of phosphorylated (i.e., more active) vs. unphosphorylated (i.e., less active) Jun and a trend toward increased levels of Fos protein in the brains of Fn14-KO mice compared to WT (Figures S3A and S3B). Together, these physiological and molecular data provide strong evidence that, particularly during a mouse’s active phase, PYR neurons are more active in the absence of Fn14.

### The circadian master regulator Bmal1 is necessary for the neuronal expression of Fn14

Most cells within the body, including neurons, express genes in a time-of-day-dependent manner through the cyclic actions of the heterodimeric transcription factors CLOCK and Bmal1. In turn, this cyclic expression poises the proteins encoded by these target genes to function most robustly during particular periods of the day. The observations that Fn14 is induced by activity within PYR cells and that Fn14 constrains PYR neuron activity according to the time of day led us to consider the possibility that Fn14 may be a gene target of the circadian clock within these neurons. Consistent with this possibility, *Fn14* expression fluctuates across the 24-h day, trending toward a peak at ZT 6 (~1:00 p.m.) compared to ZT 18 (~1:00 a.m.) (Figures 3G and 3H). Furthermore, a bioinformatic assessment^33^ of the *Fn14* gene locus identified multiple putative Bmal1 binding motifs within 1,000 bp up- and downstream of the transcription start site (TSS) for *Fn14* (Figure 3I). To directly test the hypothesis that *Fn14* expression is regulated by the molecular circadian clock in hippocampal neurons, we performed viral injections of AAV9-hSyn-Cre-eGFP into one hemisphere and AAV9-hSyn-eBFP2 (control) into the other hemisphere of Bmal1^fl/fl^ mice, a well-characterized mouse model of conditional Bmal1 loss of function, which we validated by smFISH for *Bmal1* (Figures S3C and S3D). Injections of Cre-expressing or control viruses within each hemisphere of an individual mouse allowed us to consider each mouse as an internal control, thereby limiting experimental variability. Strikingly, removal of Bmal1 from CA1 neurons at ZT 6 virtually abolished *Fn14* expression in these cells (Figures 3J and 3K). Thus, Bmal1 is a positive regulator of *Fn14* expression, indicating that *Fn14* may be a gene target of the molecular circadian clock in neurons.

### Loss of Fn14 dampens the expression of synaptic genes in PYR neurons of CA1

**Figure 4.**
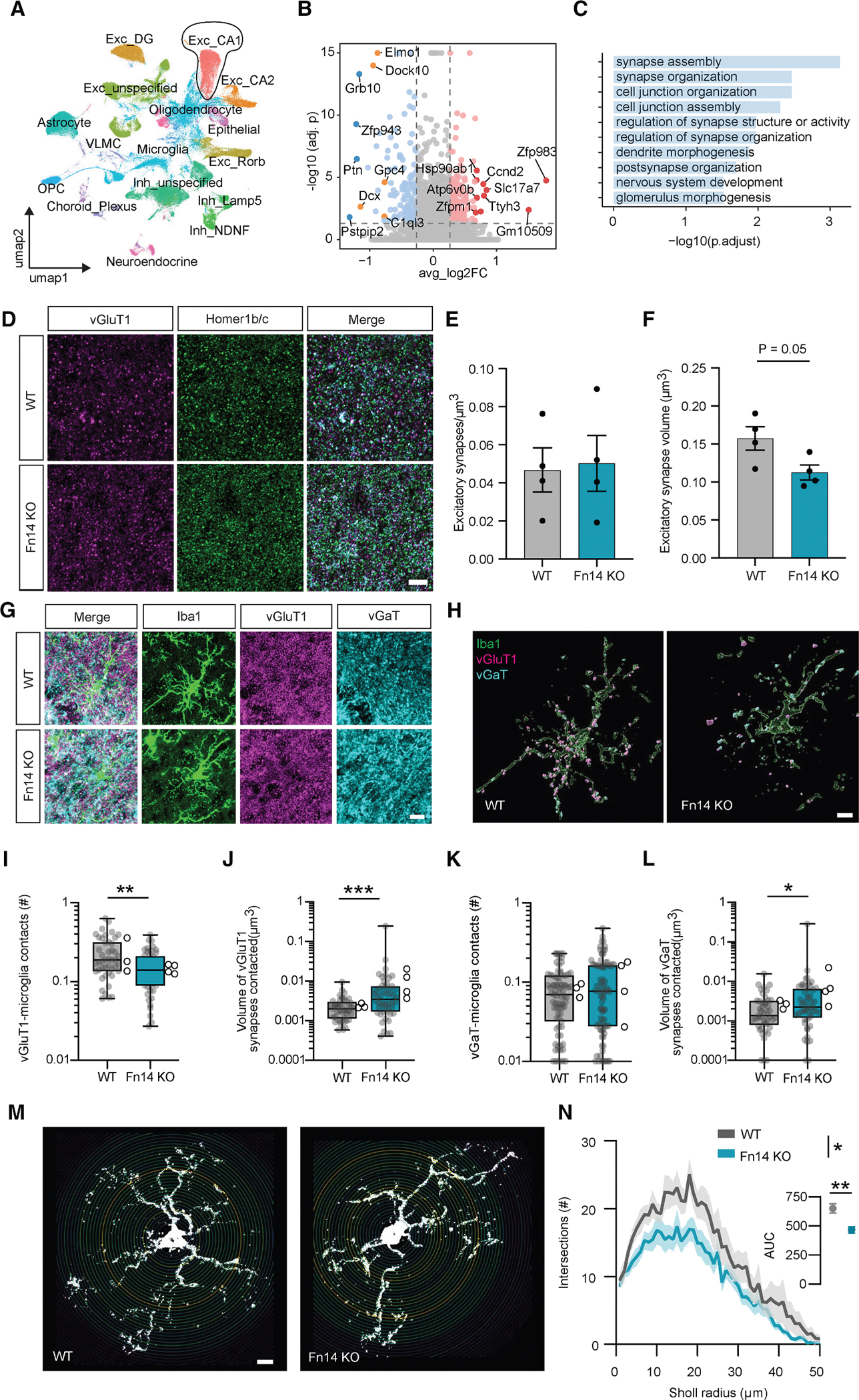
Decreased expression of synaptic genes and dysregulated microglia-excitatory synapse interactions in mice lacking Fn14 (A) Uniform manifold approximation and projection (UMAP) visualization of the hippocampal snRNA-seq dataset including cells from WT and Fn14-KO mice subjected to 24 h of environmental enrichment. The excitatory CA1 PYR neuron cluster (Exc-CA1) is circled. (B) Volcano plot showing differentially expressed genes (DEGs) in Exc-CA1 neurons between WT and Fn14-KO mice. Genes upregulated in the KO are shown in red, while downregulated genes are shown in blue. Genes of particular interest based upon their involvement in synapse assembly and organization are shown in orange. Significance thresholds were set at an average log_2_(1.2) fold change and false discovery rate (FDR) < 0.05. (C) Top 10 Gene Ontology (GO) terms enriched among DEGs downregulated in Exc-CA1 neurons from Fn14-KO mice compared to WT. (D) Confocal images of hippocampal CA1 from Fn14-KO and WT mice immunostained for the excitatory presynaptic marker vGluT1 (magenta) and the post-synaptic marker Homer1b/c (green). Rightmost column shows merge of vGluT1 and Homer1b/c with colocalizations colored in cyan. Scale bar, 5 μm. (E and F) Quantification of excitatory synapse (i.e., colocalized pre- and postsynaptic puncta) density (E) and volume (F). Data points represent individual mouse averages with mean ± SEM. Unpaired Student’s *t* test, *n* = 4 mice/genotype. (G) Representative confocal images of microglia (Iba1, green), excitatory synapses (vGluT1, magenta), and inhibitory synapses (vGaT, cyan) in WT and Fn14-KO mice. Scale bar, 10 μm. (H) Example reconstructions of microglia (Iba1, green) in contact with excitatory synapses (vGluT1, magenta) and inhibitory synapses (vGaT, cyan) in hippocampal CA1. Signals were reconstructed from fluorescence images shown in (G). (I–L) Quantification of the number and volume of excitatory synapses contacted by microglia (I and J), with quantification of the number and volume of inhibitory synapses contacted by microglia shown in (K) and (L). Data are represented on a log scale to best fit the distribution of the data. For (I)–(L), individual datapoints indicate cells, while open circles indicate mouse averages; *n* = 45/50 microglia from 3 WT/4 Fn14-KO mice. Mann-Whitney test, *p < 0.05, **p < 0.01, and ***p < 0.001. (M) Example reconstructions of microglia (Iba1) in WT and Fn14-KO mice depicting concentric rings for Sholl analysis. Scale bar, 5 μm. (N) Quantification of microglial complexity based upon Sholl analysis. Line graph depicts microglial intersections per Sholl radius with mean (dark line) ± SEM (shaded region). Repeated measures two-way ANOVA: radii, *p* < 0.0001; genotype, *p* < 0.05; interaction, *p* < 0.01. Inset plot shows area under the curve (AUC) averaged across all cells ± SEM. Unpaired Student’s *t* test, **p < 0.01; *n* = 16 cells/genotype, 3 mice/genotype. See also Figures S4 and S7.

The dynamic regulation of *Fn14* expression by both neuronal activity and Bmal1 provides insights into the mechanisms that lie upstream of Fn14 function in the brain. Through what *downstream* mechanisms might Fn14 dampen neuronal activity in CA1 following its expression? Based upon what is known about Fn14 function, we considered two main possibilities: (1) by eliciting changes in gene expression through the induction of transcriptional pathways and (2) by coordinating interactions between synapses and microglia. If Fn14 restricts neuronal activity by mediating transcription, we would expect to observe robust changes in gene expression within CA1 PYR neurons upon loss of Fn14. To test this hypothesis, we subjected Fn14-KO and WT mice to the environmental enrichment paradigm described in Figure 1 (the same paradigm that induces *Fn14* expression in CA1 neurons) and then performed single-nucleus RNA sequencing (snRNA-seq) on isolated hippocampi via a protocol that enriches for neuronal cell types.^23^ We sequenced mice following enrichment rather than at baseline to boost Fn14 expression and to home in on activity-induced transcripts. After sequencing cells across four mice per genotype, we mapped the sequencing data to the mouse genome and then subjected them to a stringent quality control pipeline that involves the removal of cells with high (2.5%) mitochondrial DNA content, dead or dying cells, putative doublets, and ambient RNA. All cells included in the dataset had between 1,000 and 30,000 unique molecular identifiers (UMIs) and 500–5,000 genes per cell. Our final dataset includes 121,376 nuclei across 17 cell types annotated based upon prior literature (Figures 4A, S4A, and S4B).

Applying differential gene expression analysis to the dataset, we identified transcriptomic differences in PYR neurons of CA1 between Fn14-KO and WT mice. However, we noticed that the fold-change effect sizes of these differences were modest, with most genes exhibiting less than a 2-fold change in expression between genotypes. This observation seems inconsistent with the idea that Fn14 mediates hippocampal function primarily by inducing transcriptional pathways. Rather, we reasoned that these relatively modest changes in gene expression could instead reflect more chronic adaptations of CA1 to a loss of Fn14. If so, characterizing the genes that are dysregulated in CA1 neurons lacking Fn14 could provide insights into the mechanisms through which Fn14 functions to influence CA1.

Thus, we next identified genes that were up- or downregulated in Fn14-KO compared to WT CA1 PYR neurons with a stringent false discovery rate (FDR < 0.05) but a less restrictive fold-change threshold of log_2_(1.2). This analysis identified 172 and 126 genes that were up- or downregulated by loss of Fn14, respectively (Figure 4B). Again, consistent with Fn14 not directly impacting gene expression, the majority of downregulated genes were not associated with transcriptional regulation but were rather associated with synapses. For example, the top three enriched Gene Ontology (GO) categories were synapse assembly, synapse organization, and cell junction organization, with dendrite morphogenesis and postsynapse organization also being significantly enriched (Figure 4C). Of particular interest, proteins that regulate actin cytoskeletal dynamics within postsynaptic structures like dendrites and spines were heavily represented among differentially expressed genes (DEGs) downregulated in the absence of Fn14. This includes signaling molecules such as the RhoGEF Dock10, which is required for dendritic spine morphogenesis, and cell-surface adhesion molecules like SynCAM1, which promote postsynaptic filopodial dynamics leading to the assembly of functional excitatory synapses^34,35^ (Figures 4B and 4C). Altogether, these data suggest that, rather than mediating hippocampal function by controlling transcription, Fn14 may influence its function by modifying synapses.

### Fn14 promotes interactions between microglia and excitatory synapses

Previous work has shown that Fn14 localizes to synapses in the brain and mediates visual circuit development by coordinating interactions between Fn14-expressing neurons and microglia, a unique population of brain-resident macrophages with known roles in synaptic remodeling.^23,36^ While microglia are widely appreciated to shape neural circuits through the phagocytic engulfment of synapses, we previously showed that they can also modify the structure and physiology of synapses through non-phagocytic mechanisms as well.^5^ To assess the possibility that Fn14 regulates hippocampal activity by recruiting microglia to modify synapses, we first asked whether loss of Fn14 altered the number or size of excitatory or inhibitory synapses in CA1. To do so, we measured the colocalization of the excitatory presynaptic marker vGluT1 with the postsynaptic marker Homer1b/c or the colocalization of the inhibitory presynaptic marker vGaT with its postsynaptic scaffold Gephyrin. While we did not observe changes in the density of either synapse type or the volume of inhibitory synapses, excitatory synapses strongly trended toward smaller volumes in the Fn14-KO mice (*p* = 0.05; Figures 4D–4F and S4C–S4E). Excitatory synapses being smaller in the absence of Fn14 is consistent with the snRNA-seq data suggesting a lower abundance of synaptic proteins in Fn14-KO mice (Figures 4B and 4C).

We next asked whether microglia could play a role in regulating the size of excitatory synapses through their physical recruitment to these inputs by Fn14. Consistent with this possibility, contact points between microglia and excitatory synapses were significantly less abundant in CA1 in the absence of Fn14 (Figures 4G–4I). Furthermore, although the average size of excitatory synapses was decreased in the absence of Fn14 overall (Figure 4F), microglia preferentially contacted excitatory synapses of higher volume in Fn14-KO compared to WT mice (Figure 4J). Conversely, the number of microglia:inhibitory synapse contacts was not altered by loss of Fn14, although the volumes of inhibitory synapses contacted by microglia were larger in the KO, similar to the result seen for excitatory synapses (Figures 4G, 4H, 4K, and 4L). Importantly, analysis of excitatory synapses and microglia in Fn14-KO and WT mice during hippocampal development between P21 and P24 revealed no differences in excitatory synapse size or synapse-microglia interactions, suggesting that these changes represent functions of Fn14 in the mature brain (Figures S5A–S5G). Moreover, a lack of systemic changes in peripheral cytokine abundance in Fn14-KO mice argues against a general decline in animal health contributing to these cellular changes (Figure S5H). Altogether, these data point toward a role for Fn14 in recruiting microglia to a cohort of relatively small excitatory synapses in CA1, with the potential outcome of modifying their structure and function. Although the numbers of inhibitory synapses and inhibitory synapse-microglia interactions were unaffected by loss of Fn14, the observation that microglia contact larger inhibitory synapses in the Fn14-KO mice may reflect an impact of Fn14 on inhibitory synapses as well.

### Fn14 gain of function in CA1 neurons elicits a rod-like microglial state and recruits microglia to dendrites and excitatory synapses

In analyzing microglia-synapse interactions, we noticed that microglia were significantly less ramified in Fn14-KO compared to WT mice (Figures 4M and 4N). This finding raised two mechanistic possibilities. First, Fn14 may function within microglia to control microglial morphology and promote microglia-synapse contacts. Conversely, neuronal Fn14 may signal non-cell-autonomously to microglia to shape their morphology and to mediate their interactions with synapses. Using smFISH, we found that *Fn14* expression was virtually undetectable in CA1 microglia (Figures S1B and S1C), whereas neurons were by far the highest expressers of *Fn14* in CA1 (Figure 1), making the second possibility more feasible than the first. Thus, we next asked whether overexpressing Fn14 in Fn14-null CA1 neurons (i.e., selectively rescuing neuronal Fn14 expression) was sufficient to reverse the changes in microglia-synapse interactions observed in KO compared to WT mice.

**Figure 5.**
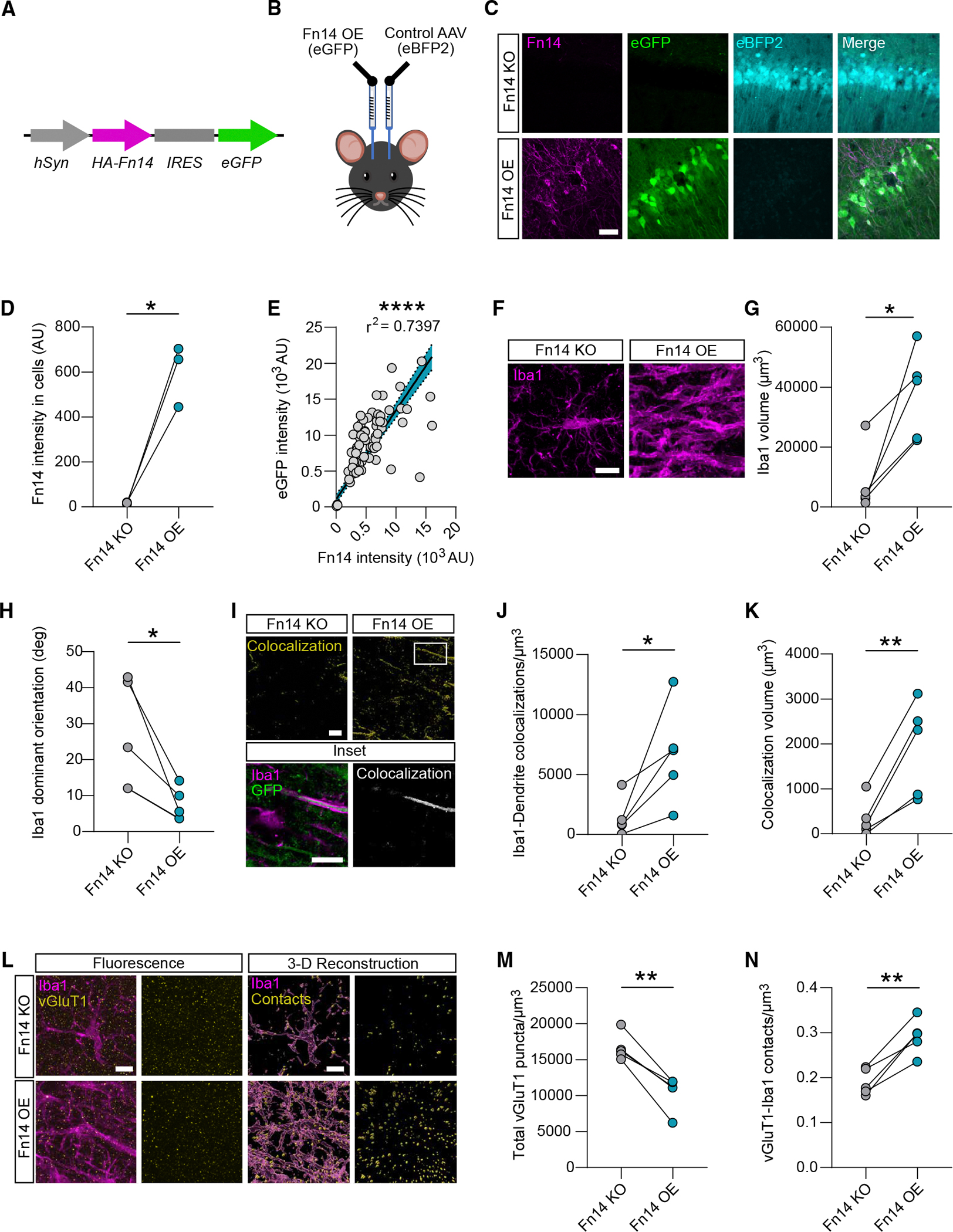
Overexpression of Fn14 in CA1 neurons induces rod-like microglia and increases their contact with dendrites and synapses (A) Schematic representation of the AAV construct for driving Fn14 overexpression (OE) in CA1 neurons. The construct consists of the human Synaptophysin promoter (hSyn, gray arrow), N-terminal HA-tagged Fn14 (HA-Fn14, magenta arrow), internal ribosome entry site (IRES, gray rectangle), and cytosolic eGFP as a marker of transduction (green arrow). (B) Illustration of experimental paradigm showing bilateral injections into CA1 of Fn14-KO mice with control AAV9-hSyn-eBFP2 into the left hemisphere and AAV9-hSyn-HA-Fn14-IRES-eGFP (i.e., Fn14 OE) into the right hemisphere. (C) Example confocal images of CA1 showing Fn14 (magenta, immunostained), eGFP (green), and eBFP2 (cyan) in control (i.e., Fn14 KO, top) and Fn14-OE (bottom) mice. Images were taken from the same animal. Scale bar, 50 μm. (D) Quantification of Fn14 fluorescence intensity in Fn14-KO and -OE hemispheres from the same mice. Paired *t* test, *p < 0.05; *n* = 3 mice, 1 hemisphere/condition/mouse. (E) Plot of eGFP by Fn14 fluorescence intensity for each cell analyzed, depicting a positive correlation. Pearson correlation coefficients, r^2^ = 0.07397, ****p < 0.0001; *n* = 122 cells. Data points show individual cells with line of best fit and 95% confidence bands (teal region). (F) Confocal images of microglia (Iba1, magenta) from Fn14-KO and Fn14-OE hemispheres within the same animal. Scale bar, 10 μm. (G) Quantification of total Iba1 volume within CA1. Data points represent individual mice. (H) Quantification of dominant angle of Iba1 signal relative to dendrites. Data points represent individual mice. (I) Colocalization channel of microglia (Iba1) and virally transduced dendrites from Fn14-KO and -OE conditions (top row). Bottom row, left: Iba1 (magenta) and eGFP (green). Bottom row, right: colocalization channel showing where Iba1 and eGFP overlap. Scale bar (top), 50 μm. Inset scale bar (bottom), 10 μm. (J and K) Quantification of total number (J) and volume (K) of Iba1-dendrite colocalizations. Data points represent individual mice. (L) Left: example confocal images of microglia (Iba1, magenta) and excitatory synapses (vGluT1, yellow) from Fn14-KO (top) and Fn14-OE (bottom) hemispheres from the same mouse. Right: 3D reconstructions of Iba1 (magenta) and vGluT1-Iba1 contacts (yellow) based upon the images on the left. (M and N) Quantification of the total number of vGluT1 puncta per image volume (M) and the number of vGluT1-Iba1 contacts per image volume (N) with data points representing individual mice. Statistical analysis for (G)–(N): paired *t* test; *p < 0.05 and **p < 0.01; *n* = 4 mice, 1 hemisphere/condition/mouse. See also Figure S5.

We generated an adeno-associated virus, AAV9-hSyn-HA-Fn14-IRES-eGFP, which expresses full-length Fn14 with an N-terminal hemagglutinin (HA) tag and simultaneously expresses cytosolic eGFP as a marker of virally transduced cells (Figure 5A). We injected this virus directly into CA1 of each Fn14-KO mouse’s left hemisphere, simultaneously injecting a control virus, AAV9-hSyn-eBFP2, into the right hemisphere, a strategy that allowed us to limit experimental variability by considering each mouse as an internal control (Figure 5B). Three weeks following injection, we harvested and sectioned each brain and validated Fn14 overexpression in eGFP^+^ (but not eBFP2^+^) CA1 neurons via immunofluorescence for Fn14 (Figures 5C–5E) and for the HA tag (Figures S6A–S6C). After validation, we performed immunofluorescence to visualize microglia and excitatory synapses in CA1 across both hemispheres. Unexpectedly, while microglia within the BFP-injected hemisphere appeared relatively normal, overexpressing Fn14 in CA1 neurons was sufficient to induce an extreme morphological state change in microglia localized to the stratum radiatum of CA1, where PYR neuron dendrites reside. Whereas classically “activated” microglia, such as those observed after brain injury or in neurodegenerative diseases, typically display an amoeboid morphology, microglia exposed to neurons that overexpress Fn14 adopted a tubular, or rod-like, structure aligned to the orientation of PYR neuronal dendrites (Figure 5F). We quantified this phenotype in three ways. First, we measured the total volume of Iba1 in the Fn14-expressing and control hemispheres, revealing a robust increase in microglial coverage after overexpression of neuronal Fn14 (Figures 5F and 5G). Second, we applied the ImageJ plug-in OrientationJ to assess the orientation of these tubular microglia relative to the dendrites of PYR neurons that overexpress Fn14. This analysis revealed that overexpressing Fn14 in CA1 neurons causes microglia to become more planar, morphologically orienting to the directionality of the dendrites (Figure 5H). These microglia not only aligned with the directionality of the dendrites but strongly colocalized with them as well (although colocalization between Iba1 and the cytosolic GFP fill from the virus may not reflect the complete range of interactions between microglia and the membranes of the dendrite; Figures 5I–5K). Thus, viral overexpression of Fn14 in CA1 neurons in an Fn14-null background elicited strong morphological changes in microglia and promoted their interactions with PYR dendrites.

We next asked whether and how overexpressing Fn14 in PYR neurons affected excitatory synapses and excitatory synapse-microglia interactions in CA1. We found that excitatory synaptic inputs were significantly less abundant in the hemisphere where Fn14 was overexpressed compared to the control hemisphere (Figures 5L and 5M). Interestingly, despite this overall decrease in vGluT1^+^ synapses, contact points between excitatory synapses and microglia were more abundant following neuronal overexpression of Fn14 (Figures 5L and 5N). Thus, viral overexpression of Fn14 in Fn14-null CA1 neurons increased the abundance of microglia-excitatory synapse interactions, a property that was decreased by global loss of Fn14 compared to WT mice. While our data indicate that these rod-like microglia may elicit a loss of neuronal structures, recent work suggests that they may play neuroprotective roles as well.^37^

### Fn14 restricts the length of the endogenous circadian period and influences sleep-wake states

To this point, our analysis has focused on the potential roles of Fn14 in hippocampal CA1. However, our use of a global Fn14-KO mouse alongside the observation that Fn14 is expressed in a plethora of brain regions raised the possibility that it may mediate adult brain function more broadly. In particular, our finding that Fn14 is regulated by Bmal1 in neurons is consistent with the idea that Fn14 may be a core regulator of circadian function beyond the hippocampus. If so, mice globally lacking Fn14 would be expected to exhibit deficits in circadian function *in vivo*.

**Figure 6.**
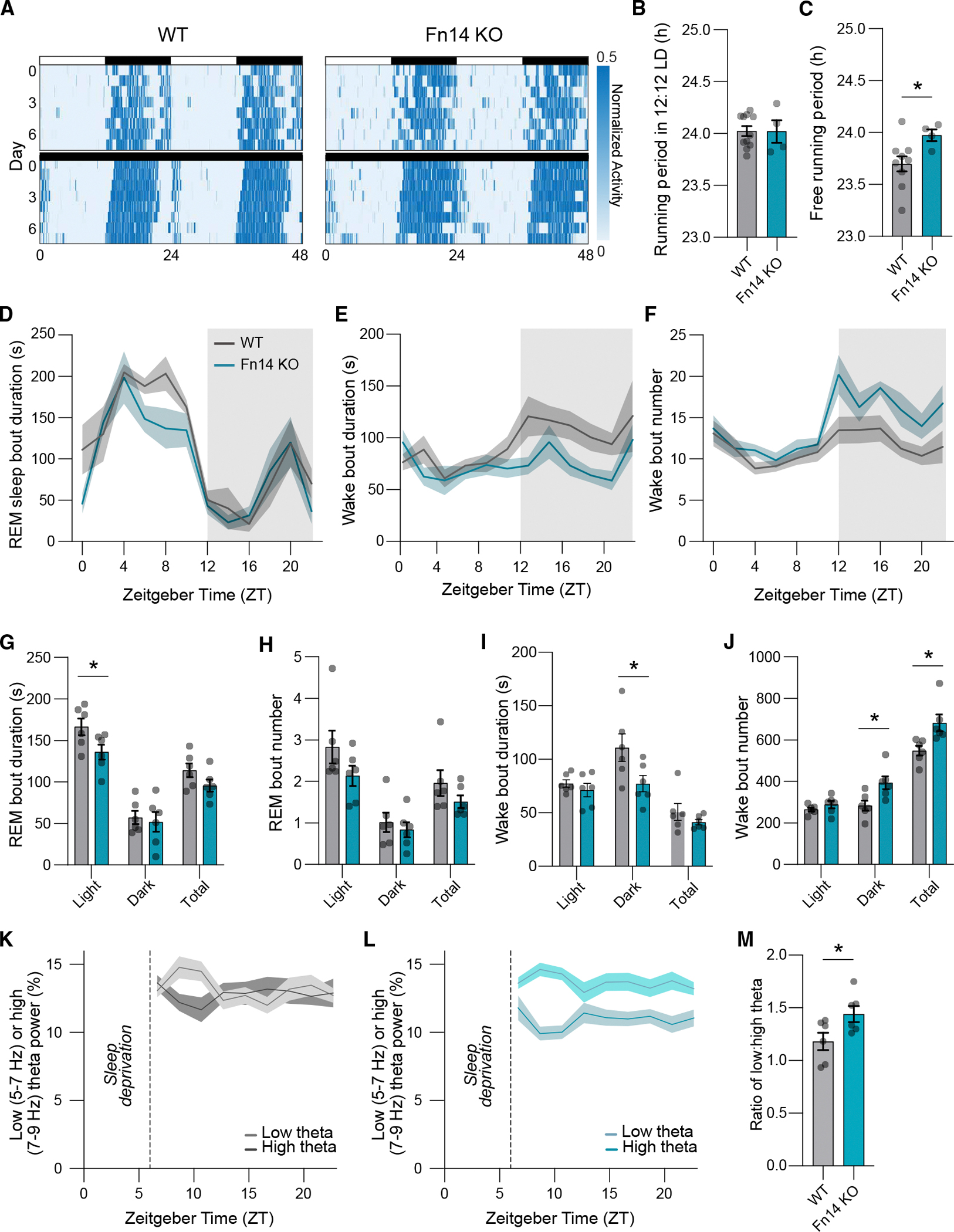
Fn14 regulates the length of the endogenous circadian period and mediates sleep-wake states *in vivo* (A) Actograms of representative WT and Fn14-KO mice under normal 12:12 light/dark conditions (top) and in constant darkness (bottom). Normalized wheel-running activity is represented in dark blue. (B) Periodicity of wheel-running activity under normal 12:12 light conditions. Unpaired Student’s *t* test, *p* > 0.05; *n* = 11 WT and 4 Fn14-KO mice. (C) Innate free-running period of Fn14-KO and WT mice during constant darkness, representative of the mouse’s internal circadian cycle. Unpaired Student’s *t* test, *p < 0.05; *n* = 11 WT and 4 Fn14-KO mice. (D–F) Quantification of electroencephalogram/electromyography (EEG/EMG) analysis of sleep and activity states during a 24-h recording period. Line, mean; shaded area, SEM. Repeated measures two-way ANOVA with Šídák’s multiple comparisons; for (D) and (E), time, genotype, and interaction: *p* > 0.05; for (F), time, *p* > 0.05, genotype, *p < 0.05, and interaction, *p* > 0.05. (G) REM bout duration (seconds) during the day (sleep phase), during the night (wake phase), and over the full 24-h period (total). (H) Quantification of bout number during the day, during the night, and over the full 24-h period. (I) Mean wake bout duration (seconds) during the day, during the night, and over the full 24-h period. (J) Mean number of wake bouts during the day, during the night, and over the full 24-h period. (K) Low (light gray) and high (dark gray) theta frequency bands following sleep deprivation in WT mice. (L) Low (light teal) and high (dark teal) theta frequency bands following sleep deprivation in Fn14-KO mice. (M) Quantification of the ratio of low and high theta frequency in WT and Fn14-KO mice. Statistics for (G)–(J) and (M): unpaired Student’s *t* test, *p < 0.05. (D)–(M) *n* = 6 mice/genotype. See also Figure S6.

Given that Fn14 constrains activity in a time-of-day-dependent manner, we asked whether circadian rhythms were altered in mice due to loss of Fn14. To this end, we employed a locomotor wheel-running assay to map active and inactive states of WT and Fn14-KO mice either in a normal 12-h/12-h light/dark cycling environment (as is found in most standard animal facilities) or in complete darkness for 24 h a day. Measuring wheel-running in mice is an established method of interrogating the behavioral output of the circadian clock. Since mice are nocturnal and run on the wheel mostly when awake, they exhibit distinct periods of wheel-running activity in a standard environment that sync up with the dark phase of the light/dark cycle. On the other hand, removing light cues allows for the unveiling of the mouse’s endogenous period (i.e., the free-running period) as light information is no longer available to entrain circadian rhythms to cues in the external environment. By measuring the wheel-running activity of Fn14-KO and WT mice under normal light/dark conditions, we found that both KOs and WTs maintained the expected 24-h circadian period (Figures 6A (top), 6B, and S6A–S6C). However, when the activity of mice was measured in constant darkness, Fn14-KO mice maintained an endogenous activity period that was significantly longer than that of their WT littermates (Figures 6A (bottom) and 6C). Thus, Fn14 may play a role in confining the length of the endogenous circadian period in mice, suggesting a role for cytokine signaling in the orchestration of internally driven oscillations that are initiated in the brain.

Circadian rhythms play an important role in brain function and whole-body physiology and are particularly critical for the regulation of oscillations in large-scale brain activity and sleep-wake states.^38,39^ Thus, we next asked how the loss of Fn14 impacts sleep and wakefulness in mice by performing chronic, wireless electroencephalogram/electromyography (EEG/EMG) telemetry with concurrent activity monitoring during normal home-cage behavior over a period of 48 h. By correlating behavioral activity with EEG/EMG data using a standardized approach,^40^ we quantified non-rapid-eye-movement (NREM) sleep characteristics, rapid-eye-movement (REM) sleep characteristics, and wakefulness in Fn14-KO and WT mice (Figures 6D–6J). While the organization of NREM sleep was normal in Fn14-KO mice, the average duration of REM sleep bouts was decreased in the absence of Fn14 (Figures 6D–6G). We also observed a trend toward a decrease in the number of REM sleep bouts in Fn14-KO mice, suggesting that mice lacking Fn14 experience less REM sleep than their WT counterparts (Figure 6H). Moreover, consistent with our finding that Fn14 constrains neuronal activity in a time-of-day-dependent manner, these decreases in REM sleep in Fn14-KO mice were restricted to the light cycle. We next evaluated the organization of waking behavior exhibited by Fn14-KO and WT mice across the recording period. We found that wake bout durations were lower in Fn14-KO mice than in WT mice during the dark phase but that the number of wake bouts was simultaneously increased, potentially in an effort to compensate for the decreased bout duration (Figures 6E, 6F, 6I, and 6J). Alongside the decrease in REM sleep experienced by mice lacking Fn14, these changes in wake bout number and duration suggest that sleep-wake states in Fn14-KO mice are, at least to some extent, fragmented.

After recording sleep-wake states in mice under normal conditions, we applied a sleep deprivation protocol to determine whether Fn14 plays a role in the re-establishment of sleep-wake patterns following forced disturbances in sleep. Briefly, we subjected mice to “gentle handling” for the first 6 h of the light cycle, when mice spend most of their time sleeping. Recovery sleep and wake data were then recorded over the subsequent 18 h (Figures S6C–S6J). Analyzing EEG/EMG data following an acute 6-h sleep deprivation protocol, we found that Fn14-KO mice exhibited higher low-to-high theta band ratios during wakefulness than WT mice during the recovery period (Figures 6K–6M). As the prevalence of low theta (5–7 Hz) to high theta (7–9 Hz) activity during wakefulness is thought to be related to sleep propensity,^41^ or the drive to attain sleep following a period of wakefulness, this result suggests that Fn14-KO mice were more tired, or fatigued, following sleep deprivation than their WT counterparts. This finding is consistent with the baseline fragmentation of sleep displayed by Fn14-KO mice (Figure 6). Overall, these data provide evidence that Fn14 influences some features of circadian biology *in vivo*.

### Loss of Fn14 increases seizure severity and seizure-related mortality *in vivo*

**Figure 7.**
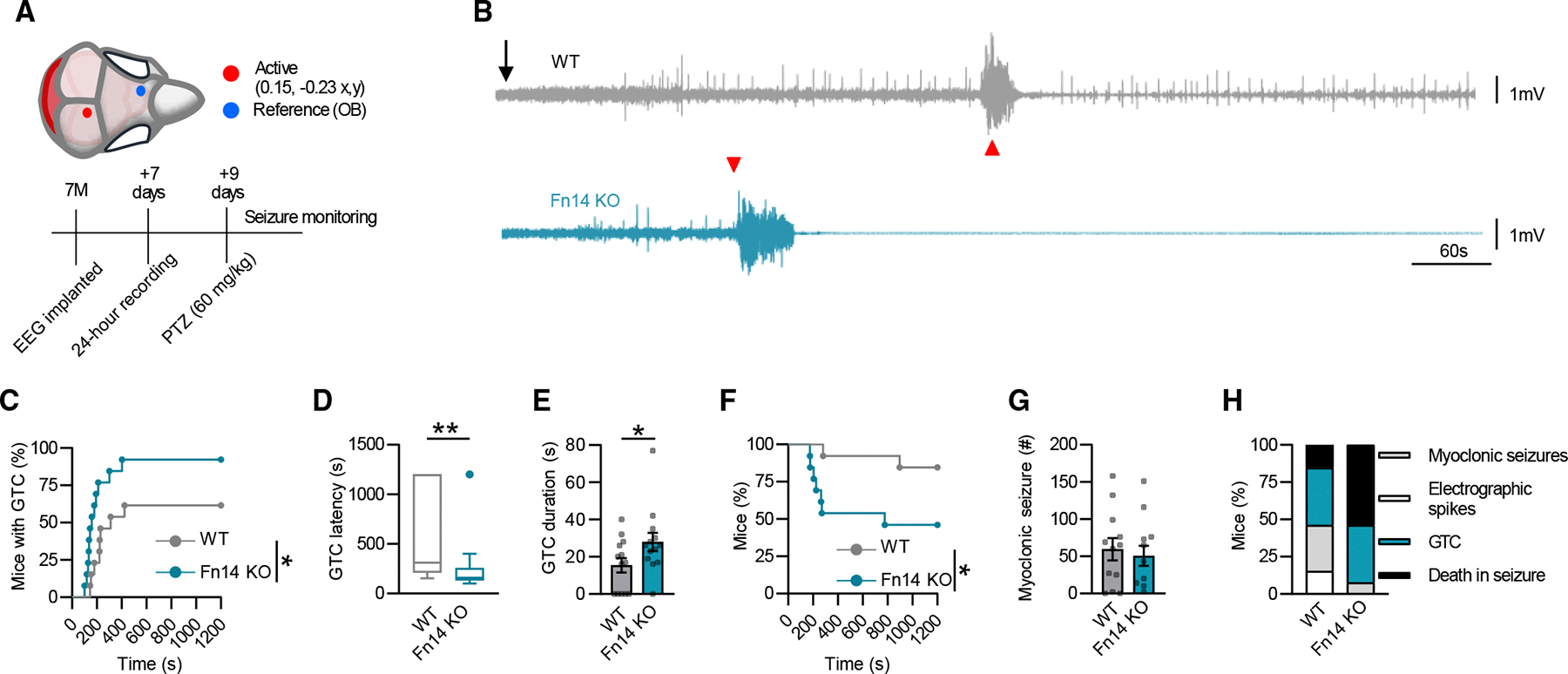
Fn14 is protective against chemically induced seizures (A) Schematic of EEG electrode placement and the experimental timeline. (B) Example EEG traces from WT (gray) and Fn14-KO (teal) mice after pentylenetetrazol (PTZ) injection (black arrow). Red triangles indicate the onset of general tonic-clonic (GTC) seizures (WT, latency = 311 s, duration = 19.8 s; Fn14 KO, latency = 159 s, duration = 35 s). The Fn14-KO mouse died shortly after GTC seizures, demonstrated by the elimination of signal following the seizure. (C) Percentage of mice that had GTC seizures relative to the time course of the experiment. WT, median = 311 s; Fn14 KO, median = 159 s. Log-rank test, *p < 0.05. (D) Latency between PTZ injection and GTC onset. Mann-Whitney test, **p < 0.01. (E) Duration of GTCs. Unpaired Student’s *t* test, *p < 0.05. (F) Mortality rate of Fn14-KO and WT mice following PTZ administration. Log-rank test, *p < 0.05. (G) Number of PTZ-induced myoclonic seizures. Mann-Whitney test, *p* > 0.05. (H) The fraction of mice presenting with electrophysiological spikes (white), myoclonic seizures (gray), GTCs (teal), or death as their worst PTZ-induced outcome. Data are presented as the mean ± SEM, with data points representing individual mice or as the percentage of subjects where applicable. (B)–(H) *n* = 13 mice/genotype.

Finally, to shed light on potential roles of Fn14 in disorders of brain function, we assessed the impact of Fn14 loss of function on seizure activity. We hypothesized that, if Fn14 is involved in dampening neuronal activity, as our data suggest, then mice lacking Fn14 would be expected to exhibit heightened sensitivity to seizures due to an inability of neurons to return to a homeostatic setpoint following activation. To test this hypothesis, we exposed Fn14-KO and WT mice to the GABA_a_ antagonist pentylenetetrazole (PTZ), a convulsant agent commonly used to elicit seizures through the dampening of inhibition onto excitatory hippocampal neurons, which we have shown to inducibly express Fn14.^42^ Intraperitoneal injection of PTZ (60 mg/kg) into Fn14-KO and WT mice time-locked with EEG recordings demonstrated profound differences in the responses of mice of each genotype to PTZ (Figure 7A). Specifically, upon PTZ injection, Fn14-KO mice were more likely than WT littermates to develop general tonic-clonic (GTC) seizures, and Fn14-KO mice developed GTC seizures at a shorter latency than WT mice (Figures 7B–7D). Furthermore, Fn14-KO mice exhibited a 152% increase in the duration of GTC seizures when compared to the GTC seizures measured in WT mice (Figure 7E). Concurrent with the marked increase in GTC severity, Fn14-KO mice had a significantly higher mortality rate after PTZ challenge compared with WT mice, with about 50% of Fn14-KO mice dying as a result of seizure induction (Figure 7F). There was no difference in the number of myoclonic seizures exhibited by Fn14-WT or -KO mice, potentially due to the higher mortality rate of Fn14-KO mice (Figure 7G). Last, loss of Fn14 led to a worse overall seizure phenotype as scored by a combination of recorded behavior, EEG activity, and mortality, suggesting that loss of Fn14 confers an increased susceptibility to acutely induced seizures that is extreme enough to cause death (Figure 7H). Altogether, these functional data reveal that loss of Fn14 exacerbates seizure severity and worsens seizure outcomes following the acute dampening of inhibition. These results are consistent with a model in which Fn14 constitutes an activity-dependent feedback loop that protects neurons from hyperexcitability by dampening their activity.

## DISCUSSION

While our results identify Fn14 as a coordinator of mature brain function, it is important to note that the TWEAK-Fn14 pathway is not the only TNF/TNFR family pathway to play a role in the brain, as TNF-α regulates circadian rhythms,^43^ activity-dependent synaptic scaling, and dendritic spine remodeling in the hippocampus.^44–47^ Furthermore, although their roles in the healthy brain are still emerging, Fn14 and its ligand TWEAK have been implicated in a diversity of diseases, including neuropsychiatric lupus, multiple sclerosis, and AD.^18,48,49^ For example, Nagy et al. found that Fn14 levels are increased in the brains of individuals with AD and that pharmacologically dampening TWEAK in hippocampal slices from a mouse model improved deficits in long-term potentiation that emerged due to pathology.^50^ In combination with these results, our finding that Fn14 influences circadian cycle length, sleep-wake states, and memory is in line with a possible role for TWEAK and Fn14 in AD. Follow-up studies will aim to determine how Fn14 transitions from homeostatic roles in the healthy adult brain toward disease states that dysregulate hippocampal circuitry.

### Limitations of the study

Several aspects of the study warrant further investigation. For example, *Fn14* is expressed in both excitatory and inhibitory neurons, yet whether Fn14 influences hippocampal function by operating within either (or both) of these cell types, or by operating within other regions that connect to the hippocampus, is not tested here. In addition, although we observed a trend toward decreased Fn14 expression as the day progresses, analysis in a larger variety of cell types at additional time points is important for substantiating these fluctuations. At the mechanistic level, although we show that Fn14 expression in CA1 neurons requires the transcription factor Bmal1, ascertaining whether Fn14 is a *bona fide* gene target of the molecular circadian clock requires orthogonal validation. Therefore, while this study uncovers numerous interesting aspects of Fn14 regulation and function, the mechanisms through which Fn14 operates in the brain remain to be fully delineated.

## RESOURCE AVAILABILITY

### Lead contact

Requests for further information or resources should be directed to and will be fulfilled by the lead contact, Lucas Cheadle (cheadle@cshl.edu).

### Materials availability

All unique reagents generated in this study are available from the lead contact with a completed material transfer agreement.

### Data and code availability

All raw and processed transcriptomic data generated in this study have been uploaded to the Gene Expression Omnibus (GEO) server with accession number GEO: GSE315059.

## STAR★METHODS

### EXPERIMENTAL MODEL AND STUDY PARTICIPANT DETAILS

All experiments were performed in compliance with protocols approved by the Institutional Animal Care and Use Committee (IACUC) at Harvard Medical School and Cold Spring Harbor Laboratory. The following mouse lines were used in the study: C57Bl/6J (The Jackson Laboratory, JAX:000664), B6.Tnfrsf12a^™1(KO)Biogen^ (Fn14 KO and WT littermates^29^), and B6.129S4(CG)-*Bmal1*^*™1Weit*^*/*J (The Jackson Laboratory, JAX:007668). Fn14 KO mice were generously provided by Dr. Linda Burkly at Biogen (Cambridge, MA) and are subject to a Material Transfer Agreement with Cold Spring Harbor Laboratory. Analyses were performed on both male and female mice between P21 and six months of age. No sex differences were observed in the study.

### METHOD DETAILS

#### Single-molecule fluorescence *in situ* hybridization (smFISH)

Sagittal or coronal sections of 20–25 μm thickness centered on the hippocampus were made using a Leica CM3050S cryostat and collected on Superfrost Plus slides then stored at −80°C until use. Multiplexed smFISH was performed using the RNAscope platform (Advanced Cell Diagnostics [ACD], Biotechne) according to the manufacturer’s instructions for fresh-frozen (multiplexed kit v1, now discontinued) or fixed-frozen (multiplexed kit v2) samples. Probes against the following transcripts were utilized: *Fn14* (*Tnfrsf12a*), *Slc17a7* (*Vglut1*), *Gad1*, *Camk2a*, *Gad2*, and *Fos*. For the quantification of *Fn14*, *Slc17a7*, and *Gad1* transcripts per cell, 60X confocal images were acquired using a LSM 710 Zeiss microscope. A total of 3 mice per condition and a minimum of two images per mouse were analyzed. *Fn14* expression was quantified using an ImageJ macro built in-house (code: www.cheadlelab.com/tools). Briefly, the DAPI channel was thresholded and binarized, and subsequently expanded using the dilate function. This expanded DAPI mask was then passed through a watershed filter to ensure that cells that were proximal to each other were separated. This DAPI mask was then used to create cell-specific ROIs, where each ROI was considered a single cell. Using these cell-masked ROIs, the number of mRNA puncta were counted using the 3D image counter function within imageJ. ROIs were classified with the following criteria: ROIs containing 3 or more *Fn14* molecules were considered positive for *Fn14*, while those containing 5 or more molecules of either *Gad1* or *Vglut1* were considered positive for each marker, respectively.

For the quantification of *Fn14*, *Camk2a*, *Gad2*, and *Fos* in mice treated with kainate or vehicle control, 40X confocal images of the hippocampus were acquired using an LSM 780 Zeiss microscope. Six images were taken per section across 4 mice per condition (kainate or vehicle [i.e. water]). Data analysis was conducted using the image processing software ImageJ (FIJI). First, a binary mask was created for the DAPI channel in each image by applying a Gaussian blur, binarizing the image, closing holes in the nuclear signal, dilating the image, and removing cells in the image that are not part of the CA1 area of the hippocampus. Using this binary mask for region of interest (ROI) analysis, the area and mean intensity were collected for each nucleus (cell) and exported into an Excel file. To calculate intensity thresholds used to determine if a cell was positive or negative for a particular marker, a supervised analysis was conducted. In this method, ten ROIs were manually selected based on if they were visually either positive or negative for a marker and their intensity values were calculated. *Gad2*, *Camk2a*, and *Fos* thresholds were extracted by collecting the mean and standard deviation of expression intensity for ROIs appearing negative for the marker and adding two standard deviations to the mean ($\bar{x}-2\sigma$) to calculate the threshold for each marker. *Fn14* intensity threshold was determined by selecting ROIs appearing positive for *Fn14* and calculating the threshold by subtracting two standard deviations of expression intensity from the mean ($\bar{x}-2\sigma$).

Next, using the thresholds created for each marker, each ROI was established as either positive (represented with 1) or negative (represented with 0) for a marker. *Fn14* intensity in cells positive or negative for a given marker, or for *Fos*, was calculated. Next, average intensity values of *Fn14* were collected from cells positive for *Camk2a* and from cells positive for *Gad2* in order to compare *Fn14* intensity across cell types. Finally, the proportion of cells co-expressing *Fn14* and each cell type marker was calculated.

For environmental enrichment, sections were imaged using a LSM780 Zeiss microscope with a 40X oil immersion objective and 1.4x digital zoom. Three images were taken per section per condition. Data analysis was conducted using ImageJ software. Images were initially processed with three rounds of the despeckle tool to remove extraneous noise followed by background subtraction with rolling ball radius set to 10 pixels. DAPI was used to designate ROIs with a minimum of 20 cells measured per brain. Puncta within each ROI were counted using the find maxima tool with prominence set to the lowest intensity of puncta within a data set (typically 600–1000). Quantifications are presented as average puncta (i.e. transcripts) per cell. Probes against the following transcripts were utilized: *Fn14* (*Tnfrsf12a;* Cat# 42931-C1), *Fos* (Cat# 316921-C3), *P2ry12* (Cat# 317601-C3), *Pdgfrα* (Cat# 480661-C2), *Olig2* (Cat# 447091-C3), and *Aldh1l1* (Cat# 405891-C2).

#### Behavior

##### Cued fear conditioning

On training day, subjects were placed into a square fear-conditioning arena of 24(w)x20(d)x30(h) cm equipped with a shock grid floor and acrylic walls patterned with horizontal black and white bars 2 cm in width. Subjects were allowed to acclimate to the arena for 4 minutes before data acquisition. During training, mice were presented with three 20 second tones (75 dB; 2000 Hz) followed by a 2 second foot shock (0.5 mA) with variable inter-trial intervals totaling 5 minutes. After training, subjects were returned to their home cages for 24 hours before being tested in familiar and novel contexts. For familiar context (the paired context without the cued tone; Context (−) tone) subjects were re-acclimated to the test arena for 5 minutes without receiving tone cues or shocks to reduce freezing to non-tone cues. After testing freezing in the Context - tone condition and on the same day, subjects were exposed to a novel context (circular arena 30(w) × 30(h) cm, with clear acrylic floor and polka-dot walls) for 3 minutes to habituate the mice to the novel context before freezing was measured. Mice were then returned to their home cages for 24 hours before being re-exposed to the novel context, then they were re-exposed to the cued tone (75 dB; 2000 Hz) for three minutes during acquisition. Freezing was calculated using Ethovision XT v. 15 (Nodulus, Netherlands*)* with activity detection set to 300 ms, and data were presented as freezing over the trial time.

##### Morris water maze

Each training trial consisted of four 90 s sub-trials in which each subject’s starting position was pseudo-randomized to each of the four cardinal directions in a 137 cm wide water bath containing 24°C clear water filled up to 25 cm from the rim of the tub. The cardinal directions were marked on the wall of the tub with 20 cm diameter symbols. Subjects were initially trained over two trials where the goal zone was visible (visible trials), where the goal platform was raised 0.5 cm above the water line and was marked with a bright flag for increased visibility. Each trial ended either after the trial time expired, or after the subject correctly found and stayed on the goal platform for more than 5 seconds. If a mouse did not find the platform within 90 seconds, it was gently moved to the platform and left there for 5 seconds. The day following visible platform training, the goal platform was submerged (0.5 cm below the water line) and moved to a different quadrant. Subjects were tested on the hidden platform over 5 consecutive trials spanning 48 hours. On the fourth day (probe trial) the goal platform was removed from the testing arena and subjects were placed facing the wall opposite of the previous goal platform’s position. Subjects were allowed to swim for a total of 60 s before being removed from the arena. On reversal trials (4 trials), the goal platform remained submerged, but was moved to the opposite end of the arena. Subjects started the reverse trials facing the furthest wall and were allowed to search for the goal platform for 90 s. If the subject failed to find the goal platform, the subject was oriented in the correct direction and guided to the goal platform before being removed from the arena. Latency to goal platform, distance swam, and subject position were collected using Ethovision XT v. 15 (Nodulus, Netherlands).

##### Optomotor testing of visual acuity

An optomotor device (CerebralMechanics, Canada) was used to measure visual acuity. The apparatus consists of 4 computer monitors arranged in a square, in order to produce a virtual 3-D environment, with a lid to enclose subjects. Using the Optomotor 1.7.7 program, a virtual cylindrical space with vertical sinusoidal gratings was drawn on the monitors such that each monitor acted as a virtual window into the surrounding cylindrical space. Mice were placed on a lifted platform in the optomotor device and allowed to move freely, and tracking software was used to position the center of the virtual cylinder at the mouse’s head. Typically, when the cylinder with the grating stimuli is rotated (12 deg/sec), mice will begin to track the grating stimuli across the virtual space with reflexive head movements in concert with the stimulus motion. If the mouse’s head tracked the cylinder rotation, it was judged that the animal could see the grating. Using a staircase procedure, the mouse was tested systematically against increasing spatial frequencies of the grating until the animal no longer responded, with the mouse’s acuity being assigned as the highest spatial frequency that the mouse responded to by tracking.

#### Fiber photometry

##### Stereotaxic surgery (viral injections and optic fiber implants)

All surgical procedures were performed in line with CSHL guidelines for aseptic technique and in accordance with the humane treatment of animals as specified by the IACUC. At the start of surgical procedures, mice were anesthetized with isoflurane (3% induction; Somnosuite, Kent Scientific), and then injected with buprenorphine SR (Zoopharm, 0.5 mg/kg, s.c.). Upon confirmation of deep anesthesia mice were placed into a stereotaxic frame (David Kopf Instruments) where they were maintained at 1–1.5% isoflurane. A midline incision was then made from the posterior margin of the eyes to the scapulae to expose the braincase. The skull was cleaned and then a drill was positioned over the skull to drill a hole for the viral injection. Mice were then injected unilaterally within the dorsal hippocampus (−2.06 mm AP, 1.3 mm ML, 1.25 mm DV) using a 30-gauge blunt Neuros syringe (Hamilton) at a rate of 20 nl/min for a total volume of 200 nL. AAV9-CamkII-GCaMP6f (viral titer 1×10^13^ gc/mL), obtained from Addgene, was injected. After the infusion, the needle was left in place for at least 10 minutes before the microinjector (World Precision Instruments) was withdrawn slowly. Directly following virus injection, a fiber optic (400 um in diameter; 0.48 NA, Doric Lenses) was lowered just dorsally to the injection site (−2.06 mm AP, 1.3 mm ML, 1.20 mm DV). The optic implant was then fixed in place with Metabond (Parkell) and dental cement. After surgery, mice were then allowed to recover until ambulatory on a heated pad, then returned to their home cage with Hydrogel and DietGel available. Mice were then allowed to recover for approximately 4 weeks to allow for viral expression before behavioral experiments and fiber photometry recordings began.

##### In vivo *optical recording*

Approximately 4 weeks after viral transduction and fiber optic implantation, baseline recording sessions began. In brief, mice were tethered to a fiber optic patch cord (400 uM, Doric Lenses) via a ceramic mating sleeve connected to the implanted optic fiber (400 uM, Doric Lenses), and fiber photometry data was collected using a fiber photometry setup with optical components from Doric Lenses and controlled by a real-time processor from Tucker Davis Technologies (TDT; RZ5P). TDT Synapse software was used for data acquisition, where LED sources of 465 nm (Signal / GCaMP) and 405 nm (Control / Isosbestic) were modulated at 211 or 230 Hz and 330 Hz, respectively. LED currents were adjusted in order to return a voltage between 100 and 150 mV for each signal, and were offset by 5 mA. The signals were then demodulated using a 6 Hz low-pass frequency filter, where subsequent analysis occurred. In brief, GuPPy, an open-source Python toolbox for fiber photometry analysis^51^, was used to compute ΔF/F and z-score values, as well as Ca^2+^ event amplitude and frequency, for all recordings. We did not analyze the first minute of each 11-minute epoch in order to remove any artifacts that may occur as the recording begins (i.e. 600 seconds was analyzed for each epoch). To calculate the change in fluorescence ΔF/F from the photometry signal F, GuPPy normalized the data by fitting the GCaMP6f signal with the isosbestic control wavelength and computing ΔF/F = Signal - Fitted Control. It then computed a standard z-score signal for the ΔF/F data using z score=F/F-(mean of F/F) standard deviation of F/F to evaluate the deviation of the ΔF/F signal from its mean. We incorporated a 600-second user-defined window for thresholding calcium transients in the ΔF/F and z-score traces; GuPPy identifies the average amplitude and frequency (defined as events per minute) of the transients in each trace, as well as the amplitude and timing of each transient. Transients were identified by filtering out events with amplitudes greater than two times the median absolute deviation (MAD) above the median of the user-defined window and finding peaks greater than three MADs above the resulting trace. We identified the maximum z-score amplitude for each epoch by finding the largest amplitude in the table of transient timestamps and amplitudes outputted by GuPPy. We used a custom Jupyter Notebook script to calculate area-under-the-curve (AUC) for ΔF/F and z-score traces in 10-minute time bins. A MATLAB script was additionally used to determine the average amplitudes of all values, all positive values, and all absolute values for the ΔF/F and z-score traces.

#### Quantification of AP-1 activation

Whole brain tissue was collected from Fn14 KO and WT mice at P27 and flash frozen in liquid nitrogen. Tissue was later thawed and homogenized in RIPA buffer (VWR) via agitation on ice for 30 minutes before centrifugation at 23,000 × *g* for 10 minutes. 5 microliters of the insoluble fraction were then diluted in Complete Lysis Buffer (Active Motif) and nuclear protein concentration was determined using a Bradford assay (Bio-rad). Once nuclear proteins were diluted to equal concentrations in Complete Lysis Buffer, 20 μg of sample was then used to quantify binding of Fos and phosphorylated Jun (P-Jun) to oligonucleotide consensus binding sites for AP-1 family members according to the manufacturer’s instructions. Briefly, nuclear extracts were added to a pre-coated 96-well plate, and antibodies against P-Jun and Fos were added and the plate was incubated for 1 hour at room temperature. After washing each well, an HRP-conjugated secondary antibody against P-Jun or Fos was added and the plate was incubated at room temperature for another hour. After washing off the unbound secondary antibody, each colorimetric reaction was developed and subsequently stopped using Stop solution. Absorbance at 450 nm was measured for protein binding within 5 minutes of addition of Stop solution with 650 nm as a reference. Technical replicates (n = 2/sample) were averaged and data was normalized to WT samples.

#### Circadian rhythms

Mice 2–3 months of age were separated and singly housed in conventional cages with the addition of wireless running wheels (Med Associates Inc: ENV-047). Mice were allowed to acclimate to their respective running wheels for 3–5 days before data acquisition. After acclimation, activity was recorded by measuring the number of running wheel rotations every minute using a wireless recording hub and associated software (Med Associates Inc: DIG-807, SOF-860). Mice were kept in normal environmental conditions within the vivarium, which is kept on a 12-hour:12-hour light/dark cycle, for 10–14 days before being placed into constant darkness for an additional 10–14 days of acquisition. After acquisition of their running wheel activity in both the 12:12 light/dark cycle and constant darkness (to record free running activity), running wheel data was parsed into these environmental conditions: 12:12 LD and constant darkness. Both datasets were then analyzed using a custom MatLab script which, in short, normalized the running wheel activity within a given mouse to the mouse’s mean running activity, and then iteratively fit sinusoidal waves to the data to find the wave with the best fit to the activity data. The period of this resultant sinusoid function was then reported as the running wheel activity period of a given mouse.

#### Wireless telemetry (sleep-wake dynamics)

Mice were deeply anesthetized under isoflurane vapors (3% induction, 1.5% maintenance) and implanted with HD-X02 biotelemetry transmitters (Data Sciences International, DSI, St. Paul, MN, USA) to allow acquisition of electroencephalogram (EEG) and electromyogram (EMG) potentials. Following immobilization in a stereotaxic apparatus, a midline incision was made extending between the caudal margin of the eyes and the midpoint of the scapulae. The skull was exposed and cleaned, and two stainless steel screws (00–96 × 1/16; Plastics One, Roanoke VA, USA) were inserted through the skull to make contact with the underlying dura mater. These screws served as cortical electrodes. One screw was placed 1 mm lateral to the sagittal suture and 1 mm rostral to Bregma. The other screw was placed contralaterally 2 mm from the sagittal suture and 2 mm caudal to Bregma. The transmitter was inserted into a subcutaneous pocket along the back of the animal. A set of leads was attached to the cortical electrodes and secured with dental cement. Another set of leads was inserted and sutured into the trapezius muscles for EMG measurement. The surgical procedures were performed using aseptic technique, and buprenorphine SR (0.05 mg/kg, SC) was administered to provide post-operative analgesia along with supplemental warmth (heating pad) until the animals were mobile. Following surgery, mice were singly housed and their cages were placed on top of receiver boards (RPC-1; DSI). These boards relay telemetry data to a data exchange matrix (DSI) and a computer running Ponemah software (version 6.1; DSI, St. Paul, MN, USA). Mice were allowed to recover from the surgery for 2 weeks prior to beginning sleep recordings.

For analysis, raw biopotentials were band pass filtered (0.3–50 Hz for EEG, and 10–100 Hz for EMG) and analyzed in 5 second epochs as previously described^40^. The delta band was set at 0.5–4.0 Hz, and the theta band was set at 6–9 Hz. Artifact detection thresholds were set at 0.4 mV for both EMG and EEG, and if >10% of an epoch fell outside this threshold, the entire epoch was scored as artifact. Wake was characterized by high frequency and low voltage EEG accompanied by high voltage EMG. NREM (i.e., slow wave sleep) sleep was characterized by low frequency and high voltage EEG (predominant delta), accompanied by low voltage EMG. REM (i.e., paradoxical) sleep was characterized by high frequency, low voltage EEG (predominantly theta) and EMG values. Five second epochs were collapsed into 1-hour bins for subsequent graphing and statistical analyses. For spectral analyses, biopotentials were visually inspected, cleaned of artifacts, and subjected to Fast-Fourier transforms. Periodogram data were collected in 5-second epochs of scored data and then the EEG power spectra for each vigilance state was compared between genotypes and at different times of day.

#### Wireless telemetry (baseline and sleep rebound recordings)

Mice were given a 24-hour acclimation period before telemetry was used to obtain EEG, EMG, body temperature, and locomotor activity continuously for 48 hours. During the first 24 hours, baseline sleep and wake data were collected and the mice were undisturbed. At the start of the next light cycle (ZT0-ZT6), mice were sleep-deprived by gentle handling for six hours^40^. Recovery sleep and wake data were then recorded over the subsequent 18 hours. All data were processed and analyzed using DSI Neuroscore software. Baseline and recovery recordings were scored as either wake, non-rapid eye movement (NREM) or rapid eye movement (REM) sleep in 5-second bins. Scorings were then analyzed in 1-hour bins for number of bouts, average bout length, and percent coverage of each sleep stage. Baseline and recovery EEG recordings were also automatically analyzed using Neuroscore for delta, theta, gamma and alpha spectral power; power density (amplitude); transitions between sleep stages; and number of microwakes (wake bouts of less than 5 seconds in duration).

#### Adeno-associated virus production

##### AAV-hSyn-eBFP2

The hSyn-eBFP2 plasmid was constructed by PCR amplification and elongation of the eBFP2 sequence from Addgene plasmid 66034 using the following PCR primers: (capitalized sequences anneal to eBFP2 whereas noncapitalized sequences are elongation sequences): eBFP2 Elongation Forward: gaaggtaccggatccgccacGATGGTGAGCAAGGGCGAG; eBFP2 ElongationReverse: tatcgataagcttgatatcgCAGCGAGTCAGTGAGCGAG. After elongation and amplification, the eBFP2 insert was assembled into Addgene plasmid 50465 after EcoRI-HF (NEB:R3101s) and BamHI-HF (NEB:R3136s) restriction digest using a Gibson reaction (NEB: E2621S). The subsequent plasmid was then packaged into an AAV9 viral vector via the Boston Children Hospital viral vector core.

##### AAV-hSyn-HA-Fn14-IRES-eGFP

The insert for an n-terminally HA-tagged full-length Fn14 (HA-Fn14) construct flanked with BamHI (n-terminal) and EcoRI (c-terminal) restriction sites was generated by GenScript. The HA-Fn14 insert was ligated into Addgene plasmid 50465 via T4 ligase (NEB:M0202S) following BamHI-HF (NEB:R3136s) and EcoRI-HF (NEB:R3101s) double digest to generate a HA-Fn14-Ires-eGFP construct. The plasmid was verified by whole plasmid sequencing via Genewiz and packaged into AAV9 vector by the Boston Children’s Hospital viral vector core.

#### Immunofluorescence

WT and Fn14 KO mice were perfused with ice cold PBS (Gibco) and 4% for paraformaldehyde (PFA), then the whole brains were harvested and post-fixed for 12 hours. After fixation, tissue was incubated in 15% and then 30% sucrose solution before being embedded in OCT (−80°C). Embedded tissue was sectioned coronally at 25 μm thickness onto Superfrost Plus slides using a Leica CM3050 S cryostat. Sections were then washed in PBS and blocked in blocking solution (PBS adjusted to 5% normal goat serum [NGS] and 0.3% Triton X-100 [TX-100]) for 1 hour at room temperature before being incubated in primary antibody solution containing Chicken anti-Iba1 (Synaptic Systems, 234 009; [1:1000]), Rabbit-anti-vGluT1 (Invitrogen YA364832 [1:1000]), and Mouse-anti-vGaT (Synaptic Systems, 131 001; [1:1000]) antibody diluted in PBS with 5% NGS and 0.1% TX-100 (probing solution), overnight at 4°C. The next day, sections were washed 3 times for 10 minutes per wash in PBS before incubation in secondary antibodies Alexafluor 488 goat anti-rabbit (Abcam 150077; [1:500]), Alexafluor 555 rabbit anti-goat (Thermofisher A21428; [1:1000]), and Alexafluor 488 chicken anti-rabbit (synaptic systems160 026; [1:1000]) diluted in probing solution for 2 hours at room temperature. Sections were then washed in PBS, covered with DAPI fluoromount-G (SouthernBiotech), and cover-slipped.

##### Microglia-synapse interactions in Fn14 KO mice

Images (40X, numerical aperture 1.4) were obtained on a LSM 780 Zeiss confocal microscope. Two sections per mouse (n = 3–4 mice/genotype) containing CA1 were imaged volumetrically to produce Z-stacks (3008 × 3008 pixels, voxel = 70.7 × 70.7 × 311 nm [x,y,z], 16-bit). Images were then converted from .CZI to .IMS files to quantify in Imaris 10.0.0, using the Imaris File Converter. A background subtraction (53.1 μm) and gaussian filter (0.0707 μm) were applied to all images under image processing in this program. Representative 3-dimensional surfaces of microglia (Iba1), vGluT1, and vGaT signals were then reconstructed in Imaris. In brief, surfaces were created using a signal intensity threshold based on the average signal intensity of a given object within the imaging field. After surfaces were created, relative distances between objects were determined and vGluT1 and vGaT puncta were then filtered and classified as being within −0.07 and 0.07 μm from a microglial surface as previously described (Lucas will add citation once I have the final doc). The stringent distance-based filter allowed us to filter out synaptic puncta that are more likely to reside within the glial cell (i.e. to have been engulfed by the cell) rather than in contact with the surface of the cell. Average values of volume and number of surface objects, denoted under “sum”, “mean”, and “count,” were exported for statistical analysis.

##### Fn14 gain-of-function studies

Mice were perfused with ice cold PBS (Gibco) and 4% for PFA, then the whole brains were harvested and post-fixed overnight in 4% PFA in PBS at 4° C. Following fixation, brains were wash 3 times for 10 mins each with PBS and sectioned at 40 μm thick using a Microm HM650V vibratome (Thermo Scientific). All subsequent steps were performed in 24 well plates with free-floating tissue. Tissue sections were blocked with 5% normal donkey serum and 0.5% TX 100 in PBS for 1 hr at room temperature with gentle agitation. Tissue was then incubated with primary antibody diluted in blocking solution overnight at 4° C with gentle agitation. Following primary incubation, sections were washed three time for 10 min in 0.1% TX 100 in PBS at room temperature. Samples were then incubated with secondary antibody diluted in blocking solution for 2 hrs at room temperature with agitation followed by three 10 min washes in 0.1% TX 100 in PBS and a final 10 min wash in PBS. Sections were mounted in Fluoromount-G and stored at 4° C until imaged. Primary antibodies used: rabbit anti-Fn14 (Cell Signaling R4403s, [1:500]), rabbit anti-HA (Cell Signaling 3724, [1:500]), chicken anti-Iba1 (Synaptic Systems 234009, [1:500]), and rabbit anti-vGluT1 (Invitrogen 48–2400, [1:500]). Secondary antibodies used: Alexafluor 555 donkey anti-rabbit (Invitrogen A31572; [1:500]) and Alexafluor 647 donkey anti-chicken (Jackson Immuno Research 703–605-155; [1:500]).

For validation of the Fn14 overexpression AAV, Z-stack images encompassing hippocampal CA1 were taken using a Zeiss LSM 710 confocal microscope with a 40X oil immersion lens. Images were then processed in ImageJ via three rounds of despeckle to remove extraneous signal followed by max intensity Z projection. Signal from CA1 cell bodies was then used to create ROIs from which fluorescence intensity measurements were taken for Fn14, HA, eGFP, and eBFP2. At least 20 cells per hemisphere were analyzed.

For Iba1 directionality assessment, Z-stack images of the stratum radiatum of CA1 were obtained on a Zeiss LSM 980 confocal microscope with Airy Scan capabilities using a 20X air objective. Images were max intensity projected in ImageJ followed by three rounds of despeckling. Images were then oriented such that PYR cell dendrites were horizontal. Iba1 directionality relative to PYR cell dendrites was then assessed using the OreintationJ plugin^52^ with the following parameters: local window 20 and orientation set to degrees. The resultant histogram was then used to determine the dominant (most often occurring) angle. For analysis, the absolute value of orientation was used to place all angles above 0.

To measure microglia-synapse interactions, images were processed and analyzed as described above for microglia-synapse interactions. Total Iba1 was used instead of individual microglia due to the inability to distinguish individual cells in the Fn14 overexpression hemisphere.

For Iba1-dendrite colocalizations, images were processed the same as for Iba1-synapse interactions. A colocalization channel between Iba1 and eGFP was then generated in Imaris software. The number and summed volume of colocalizations were then quantified in Imaris.

##### Developmental studies

Mice from Fn14 KO or WT littermate controls between P21–24 in age were euthanized and processed for IHC as described above for Fn14 gain-of-function studies. Primary antibodies used were chicken anti-Iba1 (Synaptic Systems 234009, [1:500]), rabbit anti-vGluT1 (Invitrogen 48–2400, [1:500]), and chicken anti-Homer1b/c (Synaptic Systems 160026, [1:500]). Secondary antibodies used were: Alexafluor 555 donkey anti-rabbit (Invitrogen A31572; [1:500]) and Alexafluor 647 donkey anti-chicken (Jackson Immuno Research 703–605-155; [1:500]). Brain sections were images on a Zeiss LSM 910 with Airy Scan (20X air objective). Images were processed and analyzed as described above.

#### EEG recordings and PTZ seizure induction

##### EEG telemetry unit implantation

Mice were implanted with wireless telemetry units (PhysioTel ETA-F10; DSI, Data Sciences International) under sterile techniques per laboratory protocol as described above. Under anesthesia, a transmitter was placed intraperitoneally, and electrodes were threaded subcutaneously to the cranium. After skull exposure, haemostasis, and identification of the cranial sutures bregma and lambda, two 1-mm diameter burr holes were drilled over the right olfactory bulb (reference) and left occipital cortex (active). The epidural electrodes of the telemetry units, connected to the leads of the transmitter, were placed into the burr holes, and secured using stainless steel skull screws. Once in place, the skull screws were covered with dental cement. Mice were subcutaneously injected 0 and 24 hours post-operatively with 5 mg/kg meloxicam for analgesia. After 1 week of recovery, mice were individually housed in their home cages in a 12/12 light/dark cycle, within a temperature- and humidity-controlled chamber with *ad libitum* access to food and water.

##### Baseline and PTZ seizure induction

After a 24-hour acclimation period, one-channel EEG was recorded differentially between the reference (right olfactory bulb) and active (left occipital lobe) electrodes using the Ponemah acquisition platform (DSI). EEG, core-body temperature, and locomotor activity signals were continuously sampled from all mice for 48 hours along with time-registered videos. Atvideos. At the end of baseline acquisition, all mice were provoked with a convulsive dose (60 mg/kg; i.p.) of the GABA_a_ receptor antagonist pentylenetetrazole (PTZ; Sigma-Aldrich, Co.) to measure seizure susceptibility and evaluate seizure thresholds^42,53–55^. Mice were continuously monitored for clinical and electrographic seizure activity for 20 minutes.

##### Data analysis

All data were processed and analyzed using Neuroscore software (DSI). Baseline EEG was analyzed for spontaneous seizure activity, circadian biometrics, and spectral power band analysis^53,54^. Relative spectral power in delta (1–4 Hz), theta (4–8 Hz), alpha (8–12 Hz), beta (12–30 Hz), low gamma (30–60 Hz) and high gamma (60–90 Hz) frequency bands of the baseline EEG were calculated using the fast Fourier transform (FFT) technique.

PTZ-induced seizure activity was broadly scored on a modified Raccine’s scale as electrographic spikes (score: 1), myoclonic seizures (score: 3), generalized tonic-clonic seizures (GTC; score: 5) and death (score: 6). Per mouse, number of myoclonic seizures, latency and incidence of GTC seizures, number of GTCs, and total duration of GTC were recorded. Mice without seizures were assigned a time of 20 min at the end of the PTZ challenge observation period.

#### Fn14 immunoprecipitation and Western blot analysis

Fn14 protein abundance in hippocampal lysates from mice exposed to kainate or water (vehicle) was measured by immunoprecipitating Fn14 using a rabbit anti-Fn14 antibody (Cell Signaling Technologies, 4403s) at 1:50. Briefly, hippocampi from the brains of Fn14 KO and WT littermate mice exposed to kainate or water were dissected and homogenized in buffer containing 10 mM HEPES-KOH pH 7.5, 25 mM KAc, 320 mM sucrose, 1% Triton X-100, and 250 mM NaCl, along with a complete protease inhibitor cocktail tablet (Roche) and phosphatase cocktails two and three (Sigma). Following homogenization in a 2 mL douncer, homogenates were rotated at 4°C for 10 minutes and then centrifuged at 14,000 RPM for 15 minutes at 4°C. Supernatants were cleared by rotating for 1 hour with 50 μL of protein A dynabeads (Invitrogen). Beads were collected on a magnet and supernatants transferred to a new eppendorf tube. Rabbit anti-Fn14 (4403s) was added to each sample at 1:50 and samples were rotated for 2 hours at 4°C. Samples were then rotated with 50 μL of protein A dynabeads at 4°C which were then captured on a magnet. The supernatant was discarded and the beads were washed in 1 mL of wash buffer (homogenization buffer but without sucrose) 4 times for 10 minutes/wash at 4°C. Proteins were then eluted from beads in 100 μL 1X Nupage LDS 4X Sample Buffer (Life Technologies) with 10% 2-mercaptoethanol by boiling at 95°C for one minute.

For western blotting, samples were run on 12% Bis-tris gels (Life Technologies) and transferred to 0.2 μm pore nitrocellulose (Bio-RAD). Blocking and antibody incubations were performed in TBS-T with 5% dry milk. The primary antibody used was rabbit anti-Fn14 (1:100, 4403s) and the secondary antibody was Alexafluor 647 (1:1,000). Blots were developed on a Sapphire FL Biomolecular Imager.

#### Single-nucleus RNA sequencing

##### Sample preparation and library construction

Single cell suspensions were generated from dissected hippocampi harvested from Fn14 KO or WT littermate controls following the enrichment paradigm described above. Suspensions were washed and resuspended in PBS + 0.04% BSA, and an aliquot was stained with ViaStain AOPI (Nexcelom #CS2–0106-5mL) and counted using a Countess FL II automated cell counter. Single cell suspensions were loaded into a 10X Chromium X instrument targeting 20,000 recovered cells according to the manufacturer’s instructions. Barcoding and library preparation were performed using the GEM-X Universal Gene Expression v4 kit (1000691; 10X Genomics). cDNA and libraries were checked for quality on an Agilent TapeStation, and quantified by KAPA qPCR. Libraries were sequenced on a NextSeq2000 (Illumina) using the following read format 28×10×10×90bp to an average depth of approximately 25,000 reads per cell. The Cell Ranger pipeline (v9.0.0, 10X Genomics) was used to align FASTQ files to the mouse reference genome (GRCm39–2024-A, 10X Genomics) and to produce digital gene-cell counts matrices.

##### snRNA-seq data preprocessing and decontamination

Raw FASTQ files were processed with Cell Ranger (v9.0.1, 10x Genomics) using the *refdata-gex-GRCm39–2024-A* reference, with intronic reads included to accommodate nuclear transcripts. Ambient RNA was subsequently removed with CellBender (v0.3), and the resulting denoised count matrices were repacked into HDF5 format using PyTables for downstream analyses.

##### snRNA-seq data quality control, normalization, clustering, and annotation

Filtered count matrices were processed in Seurat (v5.0) for quality control and normalization. For each sample, low-quality nuclei were excluded based on library complexity and mitochondrial content. Cells were retained if they contained 500–5,000 detected genes, 1,000–30,000 UMIs, <2.5% mitochondrial reads, and log_10_(genes/UMI) > 0.8. Doublets were identified and removed using scDblFinder, and cell cycle phase scores (S and G2/M) were computed from canonical gene sets. Normalization was performed using log-normalization (scale factor = 10,000) followed by scaling across features. All samples were processed independently with consistent parameters and subsequently merged into a unified Seurat object. Metadata fields for genotype (KO vs. WT), sex, and batch were derived from sample identifiers for downstream integration and comparative analyses. Cells were clustered using Seurat with principal component analysis (PCA), Harmony integration, and graph-based community detection. Clusters were manually annotated based on known marker genes to assign major cell types and subtypes.

##### DEG and GO enrichment analysis

For each major cell type, differential expression between KO and WT samples was assessed using Seurat (v5.0) with the MAST test, controlling for sample identity (latent.vars = “orig.ident”). Genes were considered significantly differentially expressed if they satisfied both thresholds: |log_2_(FC)| ≥ log_2_(1.2) and false discovery rate *p* < 0.05. Functional enrichment of significantly up- and down-regulated genes was performed using clusterProfiler (v4.8) with org.Mm.eg.db as the reference background, restricted to Biological Process (BP) ontology. GO terms were retained if Count ≥ 3, minGSSize = 5, maxGSSize = 2000, and adjusted *p* < 0.05. The top ten enriched terms per direction were visualized as bar plots ranked by −log_10_(*FDR*).

#### Bmal1^cKO^ loss-of-function experiments

B6.129S4(CG)-*Bmal1*^*™1Weit*^*/*J mice received bilateral hippocampal injections of AAV9-hSyn-eBFP2 and AAV9-hSyn-eGFP-Cre (Addgene 105540) in the left and right hemispheres, respectively. Stereotaxic CA1 viral injections were performed as described for fiber photometry. Animals were allowed 3 weeks for viral transduction before processing for smFISH as described in “Environmental Enrichment”. Probes against the following transcripts were utilized: *Fn14* (*Tnfrsf12a;* Cat# 429311-C1) and *Bmal1* (*Arntl;* Cat# 438741-C3). Analysis and quantification were performed as described in “Environmental Enrichment”.

#### Multiplexed analysis of cytokines

Whole blood was collected via the facial veins of Fn14 KO and WT littermates into additive-free tubes and centrifuged at 1500 g for 10 min at room temperature to isolate serum. For each mouse, 50 μL of serum were analyzed in duplicate. The cohort comprised 2 male and 2 female knockout (KO) mice and 2 female wild-type (WT) controls. Quantitative multiplexed detection of cytokines, chemokines, and growth factors was performed by Eve Technologies Corporation (Calgary, Alberta, Canada) using the Luminex^®^ 200^™^ system (Luminex Corporation/DiaSorin, Saluggia, Italy) with Bio-Plex Manager^™^ software (Bio-Rad Laboratories Inc., Hercules, California, USA). Thirty-two markers were measured in the samples using the Eve Technologies’ Mouse Cytokine/Chemokine 32-Plex Discovery Assay^®^ Array (MD32) as per the manufacturer’s instructions for use (MILLIPLEX^®^ Mouse Cytokine/Chemokine Magnetic Bead Panel Cat. #MCYTOMAG-70K, MilliporeSigma, Burlington, Massachusetts, USA). The 32-plex array measured the following factors: Eotaxin/CCL11, G-CSF/CSF-3, GM-CSF, GROα/CXCL1/KC/CINC-1, GROβ/CXCL2/MIP-2/CINC-3, IFN-γ, IL-1α, IL-1β, IL-2, IL-3, IL-4, IL-5, IL-6, IL-7, IL-9, IL-10, IL-12(p40), IL-12(p70), IL-13, IL-15, IL-17, IP-10/CXCL10, LIF, LIX, MCP-1/CCL2, M-CSF, MIG/CXCL9, MIP-1α/CCL3, MIP-1β/CCL4, RANTES/CCL5, TNF-α and VEGF-A. Assay sensitivities of these markers ranged from 0.3 – 30.6 pg/mL. Individual analyte sensitivity values are available in the MilliporeSigma MILLIPLEX^®^ protocol.

### QUANTIFICATION AND STATISTICAL ANALYSIS

All statistics and n numbers are described in the figure legends. For all analyses, sample sizes were chosen based on previously generated data and power analyses. Acquired data was first tested for normality and log-normality before choosing a parametric or non-parametric statistical test. When the data were found to be normal, parametric t-tests, one-way ANOVAs, or repeated measures two-way ANOVAs were used. If data was found to be non-gaussian and non-logarithmic, a Mann-Whitney test was performed.

Statistical analyses were performed in Excel and Prism 9.0 (GraphPad Software). Figures were created using MATLAB R2019b and Graphpad Prism and formatted using Adobe Illustrator (2024). Data are presented as mean ± SEM unless otherwise indicated.

## Supplementary Material

1

SUPPLEMENTAL INFORMATION

Supplemental information can be found online at https://doi.org/10.1016/j.celrep.2026.116926.

## Figures and Tables

**KEY RESOURCES TABLE:** 

REAGENT or RESOURCE	SOURCE	IDENTIFIER

Antibodies		

Chicken anti Iba1	Synaptic Systems	Cat# 234009; RRID: AB_2891282
Rabbit anti vGluT1	Invitrogen	Cat# 48-2400; RRID: AB_2533843
Mouse anti vGaT	Synaptic Systems	Cat# 131001; AB_2315584
Alexafluor 488 goat anti rabbit	Abcam	Cat# 150077; RRID: AB_2630356
Alexafluor 555 rabbit anti goat	Thermofisher	Cat# A21428; RRID: AB_2535849
Alexafluor 488 chicken anti rabbit	Synaptic Systems	
Rabbit anti Fn14	Cell Signaling	Cat# 4403s; RRID: AB_10693941
Rabbit anti HA	Cell Signaling	Cat# 3724; RRID: AB_1549585
Rabbit anti vGluT1	Invitrogen	Cat# 48-2400; RRID: AB_2533843
Alexafluor 555 donkey anti rabbit	Invitrogen	Cat# A31572; RRID: AB_162543
Alexafluor 647 donkey anti chicken	Jackson Immuno Research	Cat# 703-605-155; RRID: AB_2340379
Chicken anti Homer1b/c	Synaptic Systems	Cat# 160026; RRID: AB_2713982

Bacterial and virus strains		

pENN.AAV.CamKII.GCaMP6f.WPRE.SV40	James M. Wilson	Addgene 100834
eBFP2 (eBFP2)_CD	Iverson et al 2016	Addgene 66034
pAAV-hSyn-EGFP	Bryan Roth	Addgene 50465
AAV9-hSyn-eBFP2	Made in house	NA
AAV9-hSyn-HA-Fn14-IRES-eGFP	Made in house	NA
pENN.AAV.hSyn.HI.eGFP-Cre.WPRE.SV40	James M. Wilson	Addgene 105540

Chemicals, peptides, and recombinant proteins		

Kainate	Sigma-Aldrich	Cat# K0250-50MG
EcoRI-HF	New England BioLabs	Cat# NEB:R3101s
BamHI-HF	New England BioLabs	Cat# NEB:R3136s
Gibson reaction	New England BioLabs	Cat# NEB: E2621S
T4 ligase	New England BioLabs	Cat# NEB:M0202S
Pentylenetetrazole	Sigma-Aldrich	Cat# P6500
ViaStain AOPI	Nexcelom	Cat# CS2-0106-5mL
RIPA lysis buffer	Thermoscientific	Cat# 89900
Protease inhibitor cocktail	Thermoscientific	Cat# 78429
Phosphotase inhibitor cocktail	Thermoscientific	Cat# 78428
Sodium n-dodecyl sulfate (SDS)	Thermoscientific	Cat# 28364
1M Tris-HCI, pH 8.0	Thermoscientific	Cat# 15568025
NuPAGE^™^ LDS Sample Buffer (4X)	Thermoscientific	Cat# NP0007
XT MES Running Buffer	Bio-Rad	Cat# 1610789
Sodium Deoxycholate Detergent	Thermoscientific	Cat# 89904

Critical commercial assays		

RNAscope multiplex fluorescent V2 assay	Advanced Cell Diagnostics [ACD], Biotechne	Cat# 323110
Quantitative multiplexed detection of cytokines, chemokines, and growth factors	Eve Technologies Corporation	NA
GEM-X Universal Gene Expression v4 kit	10X Genomics	Cat# 1000691
TransAM^®^ AP-1 Family	Activ Motif	Cat# 44296

Deposited data		

Pollina et al., (2023) RNA sequencing data	Pollina et al.^27^	GEO ID# GSE175965
Yap et al., (2021) RNA sequencing data	Yap et al.^28^	GEO ID# GSE158843
Single nuclear RNA sequencing data	This paper	GEO accession# GSE315059
Yap et al., (2021) RNA sequencing data	Yap et al.28	GEO ID# GSE158843

Experimental models: Organisms/strains		

Mouse:C57Bl/6J	The Jackson Laboratory	Cat# JAX:000664
Mouse:B6.Tnfrsf12a^tm1(KO)Biogen^	Dr. Linda Burkly, Biogen	NA
mouse:B6.129S4(CG)-*Bmal1^tm1Weit^*/J	The Jackson Laboratory	Cat# JAX:007668

Oligonucleotides		

gaaggtaccggatccgccacGAT GGTGAGCAAGGGCGAG	Millipore Sigma	NA
tatcgataagcttgatatcg CAGCGAGTCAGTGAGCGAG	Millipore Sigma	NA
ACAAAAGCTAGCgccaccAT GTACCCATACGATGTTCCAGAT TACGCTAtggcttcggcttggccgc ggtctctgccgcagatcctcgtg ttgggattcggcttggtgttgatgcgcgccg cggccggggagcaagcaccaggcacctc cccatgctctagcggcagctcctggagcgcg gacctcgacaagtgcatggactgcgcttctt gtccagcgcgaccacacagcgac ttctgcctgggatgcgccgcagcacctcctgcccac ttcaggctactgtggcccattctg gggggcgctcttagtctggtcctggttttgg cgctggtttctagtttcctggtctggagaagatgc cgccggagagaaaagtttactacccccat agaggagactggtggagagggctgcc caggtgtggcactgatccagtgaccattaacgcgc gaccagcttgatatcgaattggcccct ctccctcccccccccctaacgtta ctggccgaagccgcttggaataa ggccggtgtgcgtttg tctatatgttattttccaccatattgc cgtcttttggcaatgtgagggcccggaa acctggccctgtcttcttgacgagcattcctaggg gtctttcccctctcgccaaaggaatg caaggtctgttgaatgtcgtgaagg aagcagttcctctggaagcttcttga agacaaacaacgt ctgtagcgaccctttgcaggcagcg gaaccccccacctggcgacaggt gcctctgcggccaaaagccacgtg tataagatacacctgcaa aggcggcacaaccccagtgcca cgttgtgagttggatagttgtggaaaga gtcaaatggctctcctcaagcgtatt caacaaggggctga aggatgcccagaaggtaccccattg tatgggatctgatctggggcctcggtaca catgctttacatgtgtttagtcgaggttaaaaaaac gtctaggccccccgaaccacggggac gtggttttcctttgaaaaacacgatgataat atggccacaaccatggtgagcaagggcgaggag ctgttcaccggggtggtgcccatcc tggtcgagctggacggcgacgtaaa cggccacaagttcagcgtgtccggcg agggcgagggcgat gccacctacggcaagctgaccctga agttcatctgcaccaccggcaagctgc ccgtgccctggcccaccctcgtga ccaccctgacctac ggcgtgcagtgcttcagccgctacccc gaccacatgaagcagcacgacttcttc aagtccgccatgcccgaaggctac gtccaggagcgc accatcttcttcaaggacgacggca actacaagacccgcgccgaggtg aagttcgagggcgacaccctggtga accgcatcgagctgaag ggcatcgacttcaaggaggacggc aacatcctggggcacaagctggag tacaactacaacagccacaacgt ctatatcatggccgacaag cagaagaacggcatcaaggtgaa cttcaagatccgccacaacatcgag gacggcagcgtgcagctcgccgac cactaccagcagaacacc cccatcggcgacggccccgtgctg ctgcccgacaaccactacctgagca cccagtccgccctgagcaaagacc ccaacgagaagcgcgat cacatggtcctgctggagttcgtgacc gccgccgggatcactctcggcatgg acgagctgtacaagtaatagGGC GCGCCACAAAA	GeneScript	NA

Software and algorithms		

FIJI	Schindelin et al 2012	NA
Imaris	Oxford Instruments	NA
TDT Synapse	Tucker Davis Technologies	NA
GuPPy	Sherathiya et al 2021	NA
MATLAB	MathWorks	NA
DSI Neuroscore	Data Science International	NA
Cell Ranger	10x Genomics	NA
CellBender	Broad Institute	NA
Seurat	Satija Lab	NA
clusterProfiler	Yu Lab	NA
Wheel Manager Data Acquisition Software	Med Associates Inc	Cat# SOF-860
Ponemah software	Data Science International	NA
Prism	GraphPad	NA

Other		

RNAscope probe *Tnfrsf12a*	Advanced Cell Diagnostics [ACD], Biotechne	Cat# 42931-C1
RNAscope probe *Fos*	Advanced Cell Diagnostics [ACD], Biotechne	Cat# 316921-C3
RNAscope probe *P2ry12*	Advanced Cell Diagnostics [ACD], Biotechne	Cat# 317601-C3
RNAscope probe *Pdgfrα*	Advanced Cell Diagnostics [ACD], Biotechne	Cat# 480661-C2
RNAscope probe *Olig2*	Advanced Cell Diagnostics [ACD], Biotechne	Cat# 447091-C3
RNAscope probe *Aldh1l1*	Advanced Cell Diagnostics [ACD], Biotechne	Cat# 405891-C2
RNAscope probe *Slc17a7*	Advanced Cell Diagnostics [ACD], Biotechne	Cat# 415611-c2
RNAscope probe *Camk2a*	Advanced Cell Diagnostics [ACD], Biotechne	Cat# 445231-c2
RNAscope probe *Gad2*	Advanced Cell Diagnostics [ACD], Biotechne	Cat# 415621-c2
RNAscope probe *Fos*	Advanced Cell Diagnostics [ACD], Biotechne	Cat# 316921-C3
RNAscope probe *P2ry12*	Advanced Cell Diagnostics [ACD], Biotechne	Cat# 317601-C3
RNAscope probe *Pdgfrα*	Advanced Cell Diagnostics [ACD], Biotechne	Cat# 480661-C2
RNAscope probe *Olig2*	Advanced Cell Diagnostics [ACD], Biotechne	Cat# 447091-C3
RNAscope probe *Aldh1l1*	Advanced Cell Diagnostics [ACD], Biotechne	Cat# 405891-C2
RNAscope probe *Slc17a7*	Advanced Cell Diagnostics [ACD], Biotechne	Cat# 416631-c2
RNAscope probe *Gad1*	Advanced Cell Diagnostics [ACD], Biotechne	Cat# 400951-c2
RNAscope probe *Gad2*	Advanced Cell Diagnostics [ACD], Biotechne	Cat# 439371-c2
RNAscope probe *Fos*	Advanced Cell Diagnostics [ACD], Biotechne	Cat# 316921-C3
RNAscope probe *P2ry12*	Advanced Cell Diagnostics [ACD], Biotechne	Cat# 317601-C3
RNAscope probe *Pdgfrα*	Advanced Cell Diagnostics [ACD], Biotechne	Cat# 480661-C2
RNAscope probe *Olig2*	Advanced Cell Diagnostics [ACD], Biotechne	Cat# 447091-C3
RNAscope probe *Aldh1l1*	Advanced Cell Diagnostics [ACD], Biotechne	Cat# 405891-C2
RNAscope probe *Slc17a7*	Advanced Cell Diagnostics [ACD], Biotechne	Cat# 415611-c2
RNAscope probe *Camk2a*	Advanced Cell Diagnostics [ACD], Biotechne	Cat# 445231-c2
RNAscope probe *Gad2*	Advanced Cell Diagnostics [ACD], Biotechne	Cat# 415621-c2
RNAscope probe *Fos*	Advanced Cell Diagnostics [ACD], Biotechne	Cat# 316921-C3
RNAscope probe *P2ry12*	Advanced Cell Diagnostics [ACD], Biotechne	Cat# 317601-C3
RNAscope probe *Pdgfrα*	Advanced Cell Diagnostics [ACD], Biotechne	Cat# 480661-C2
RNAscope probe *Olig2*	Advanced Cell Diagnostics [ACD], Biotechne	Cat# 447091-C3
RNAscope probe *Aldh1l1*	Advanced Cell Diagnostics [ACD], Biotechne	Cat# 405891-C2
RNAscope probe *Slc17a7*	Advanced Cell Diagnostics [ACD], Biotechne	Cat# 416631-c2
RNAscope probe *Gad1*	Advanced Cell Diagnostics [ACD], Biotechne	Cat# 400951-c2
RNAscope probe *Gad2*	Advanced Cell Diagnostics [ACD], Biotechne	Cat# 439371-c2

## Highlights

- Neuronal activity induces Fn14 expression in PYR neurons of hippocampal CA1
- Fn14 dampens PYR neuron activity in a time-of-day-dependent manner
- Fn14 expression in neurons is regulated by the circadian master regulator Bmal1
- Neuronal Fn14 induces rod-like microglia and recruits them to excitatory synapses
