# Supplementary material for: Fn14 is an activity-dependent, Bmal1-regulated cytokine receptor that induces rod-like microglia and restricts neuronal activity in vivo
Source: Cell Rep. Author manuscript; Available in PMC 2026 Apr 8. (PMC13058834; doi:10.1016/j.celrep.2026.116926)
Supplement: 1 [file NIHMS2151715-supplement-1.pdf]

Cell Reports, Volume *45*

Supplemental information

Fn14 is an activity-dependent, Bmal1-regulated cytokine receptor that induces rod-like microglia and restricts neuronal activity *in vivo*

Austin Ferro, Dominic J. Vita, Trevor Fallon, Anosha Arshad, Leah Boyd, Tess Stanley, Qianyu Lin, Adrian Berisha, Uma Vrudhula, Adrian M. Gomez, Irene Sanchez-Martin, Jeremy C. Borniger, and Lucas Cheadle

Figure S1 (Related to Figure 1). Activity-dependent expression of *Fn14* in the hippocampus.

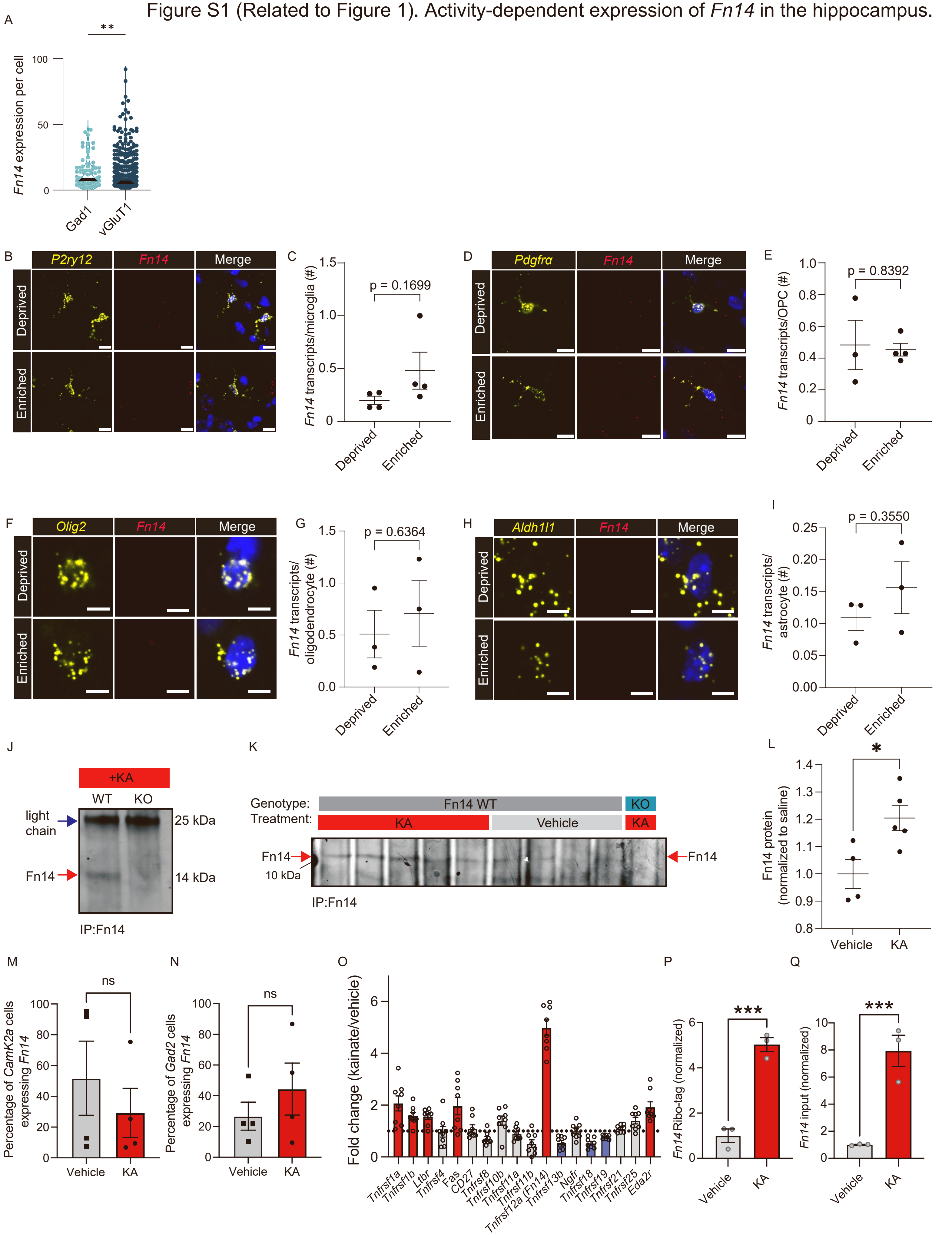

Figure S1 (Related to Figure 1). Activity-dependent expression of *Fn14* in the hippocampus. (A) Quantification of *Fn14* expression per cell in *Gad1*+ or *vGluT1*+ cells. Unpaired Student's *t*-test, **p < 0.001; n = 81 *Gad+/Fn14*+ cells and 1066 *vGluT1+/Fn14*+ cells. (B),(D),(F),(H) Confocal images of CA1 in deprived versus environmentally enriched animals showing smFISH for *Fn14* (red) in four glial cell types (yellow): microglia (*P2ry12*, [B]), OPCs (*Pdgfrα*, [D]), oligodendrocytes (*Olig2*, [F]), and astrocytes (*Aldh1l1*, [H]). (C),(E),(G),(I) Quantification of *Fn14* expression in glial cells in deprived versus enriched mice. Unpaired Student's *t*-tests, p-values displayed on graphs. Data points represent individual mice showing mean ± S.E.M. (C) n = 4 deprived and 4 enriched mice; (E) n = 3 deprived, 4 enriched; (G) n = 3 deprived, 3 enriched; (I) n = 3 deprived, 3 enriched. (J) Western blot of hippocampal lysates subjected to immunoprecipitation (IP) for Fn14 following kainic acid (KA) exposure in WT and Fn14 KO mice. Blue arrow indicates light chain from antibody, red arrow indicates Fn14. (K) Western blot of Fn14 IP in hippocampal lysates from WT mice receiving KA (left) or water as vehicle control (right). Far right lane shows IP from an Fn14 KO mouse after KA exposure as negative control. (L) Quantification of (K) normalized to control. Unpaired Student's *t*-test, *p < 0.05; n = 4 mice (vehicle) and 5 mice (KA). Data points represent individual mice, mean ± S.E.M. (M),(N) Quantification of the percentage of *Camk2a*+ PYR neurons (M) or *Gad2*+ inhibitory neurons (N) in CA1 that express *Fn14* in response to KA. Unpaired Student's *t*-test, p > 0.05; n = 4 mice/condition. Data points are individual mice, mean ± S.E.M. (O) Fold change in expression of genes encoding Tumor Necrosis Factor Receptor Superfamily members in the hippocampus after exposing mice to KA for two hours, reanalyzed based upon Pollina et al., 2023. Dashed line = 1 (no change). Red bars, genes that were significantly upregulated by KA exposure; blue bars, genes that were downregulated by KA exposure; gray, genes that were unchanged by KA exposure. (P) Normalized quantification of Fn14 expression in RNA extracted from PYR neurons, replotted from Yap et al., 2023. Unpaired Student's *t*-test, ***p<0.001; n = 3 mice/condition. (Q) Normalized quantification of Fn14 expression in the input fraction from the same experiment. Unpaired Student's *t*-test, ***p<0.001; n = 3 mice/condition.

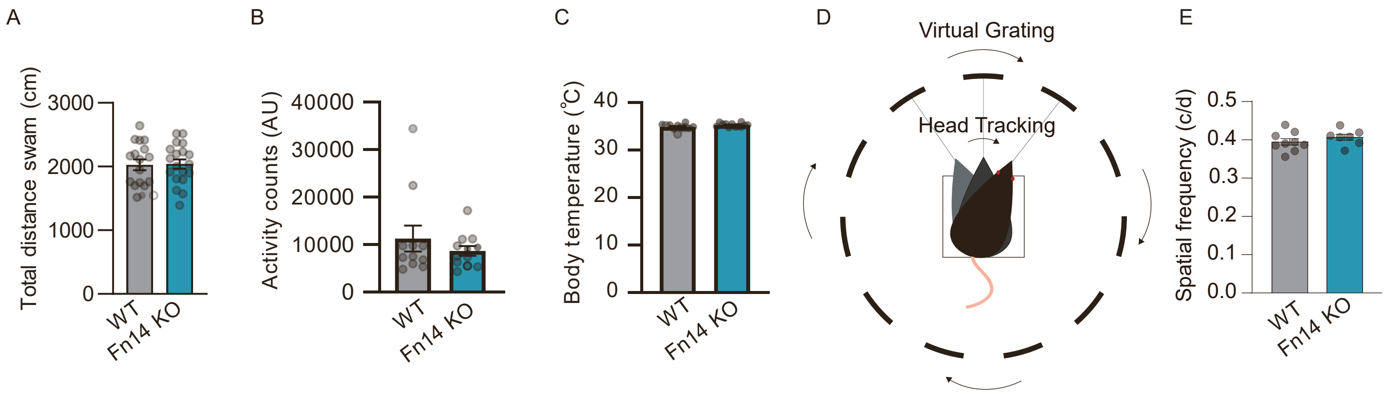

Figure S2 (Related to Figure 2). Visual acuity and locomotor activity are normal in the absence of Fn14.

Figure S2 (Related to Figure 2). Visual acuity and locomotor activity are normal in the absence of Fn14. (A) Total distance swam by mice during the Morris Water Maze probe trial. (B) Overall activity levels in Fn14 KO and WT mice measured by automated detection in video recordings during the EEG experiments. (C) Body temperatures were largely equivalent in Fn14 KO and WT mice. Unpaired Student's *t*-test, $p > 0.05$. (D) Schematic of the optomotor task in which four monitors surrounding the mouse create a virtual visual grating with varying spatial frequency. When the grating is perceptible, the mouse will track the grating with a stereotyped head movement. (E) Quantification of visual acuity in Fn14 WT and KO mice. Unpaired Student's *t*-test, $p > 0.05$; n = 9 WT and 7 Fn14 KO mice. (A)-(C) n = 17 WT and 19 Fn14 KO mice; (E) n = 9 WT and 7 Fn14 KO.

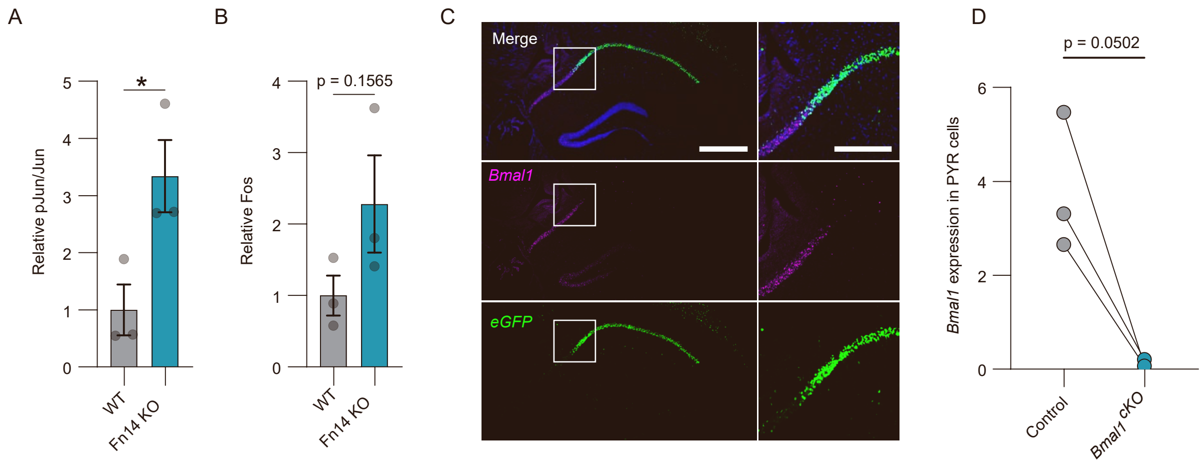

Figure S3 (Related to Figure 3). AP1 transcription factor activity is increased in the brains of Fn14 KO mice and validation of ablation of Bmal1 in Bmal1cKO mice.

Figure S3 (Related to Figure 3). AP1 transcription factor activity is increased in Fn14 KO mice and validation of Bmal1 ablation in Bmal1^{cKO} mice. (A) ELISA quantification of the relative amount of phosphorylated Jun versus unphosphorylated Jun in brain homogenates normalized to WT. Unpaired Student's *t*-test, *$p < 0.05$. (B) ELISA quantification of relative Fos protein concentration in brain homogenates normalized to WT. Unpaired Student's *t*-test, $p = 0.1565$. For (A) and (B), $n = 3$ mice/genotype. (C) Representative smFISH confocal images of the hippocampus from a *Bmal1*$^{fl/fl}$ mouse injected with an AAV-Cre-IRES-eGFP virus into CA1. *Bmal1* (magenta) and e*GFP* (green) are shown. Scale bar, 100 μm. Inset scale bar, 50 μm. (D) Quantification of *Bmal1* mRNA in control (i.e. eBFP2 transduced) versus *Bmal1*^{cKO} (i.e. Cre transduced) hemispheres. Paired *t*-test, $p = 0.0502$; $n = 3$ mice. Data points are individual mice.

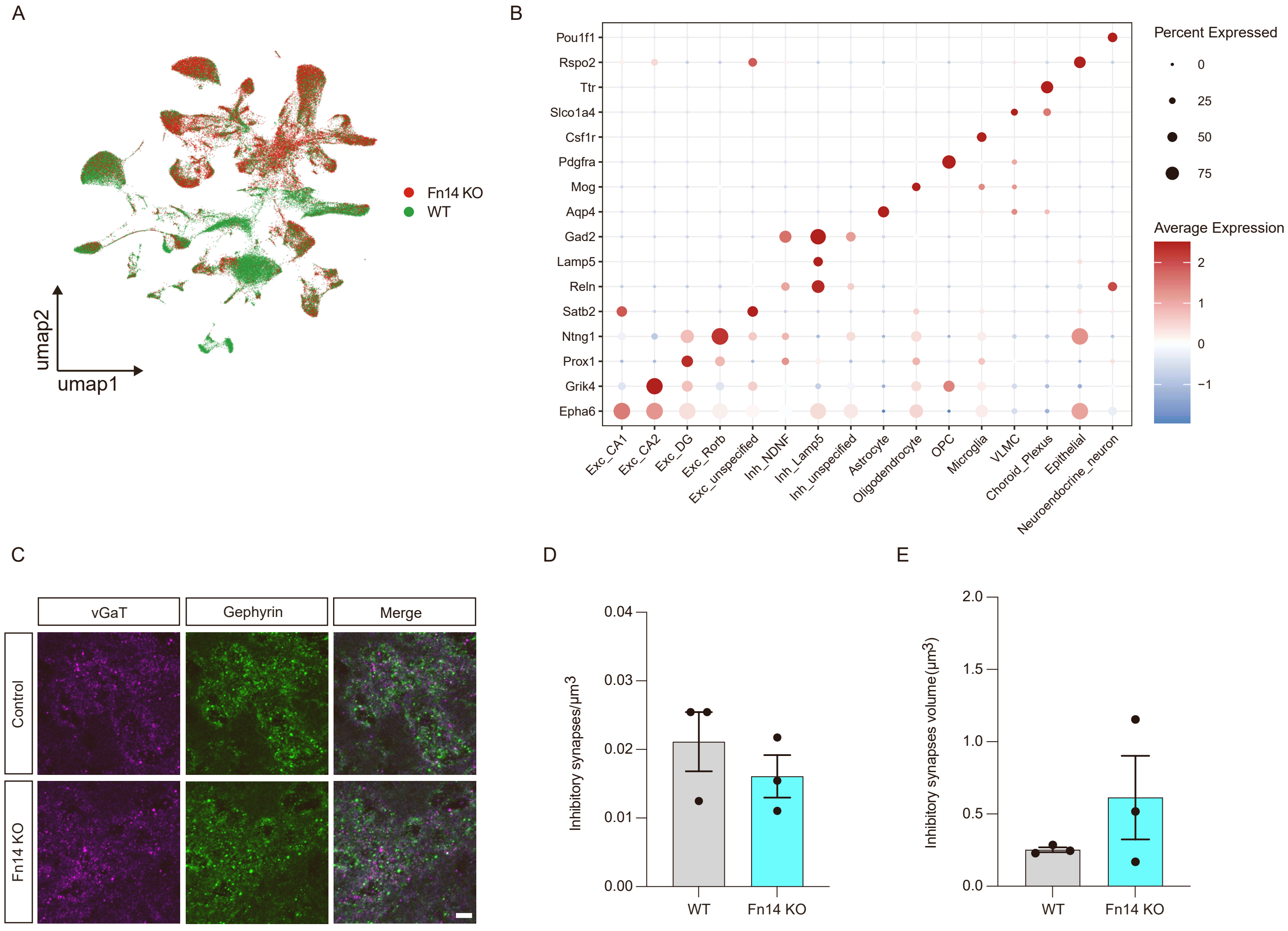

Figure S4 (Related to Figure 4). Single-nucleus RNA-sequencing cell annotations and impact of Fn14 ablation on inhibitory synapses.

Figure S4 (Related to Figure 4). Single-nucleus RNA-sequencing cell annotations and impact of Fn14 ablation on inhibitory synapses. (A) UMAP visualization of snRNA-seq data from Fn14 KO and WT mice following environmental enrichment paradigm, with cells colored by genotype. (B) Plot of cell type-specific marker expression used for major cell type annotation. (C) Confocal images of inhibitory pre- and postsynaptic markers vGaT (magenta) and Gephyrin (green), respectively, in WT and Fn14 KO mice. Scale bar, 2 μm. (D),(E) Quantification of vGaT-Gephyrin colocalization puncta (i.e. inhibitory synapses) per image volume (D) and average volumes of inhibitory synapses (E). Unpaired Student's *t*-test, $p > 0.05$; $n = 3$ mice/genotype. Data points depict individual mice with mean ± S.E.M.

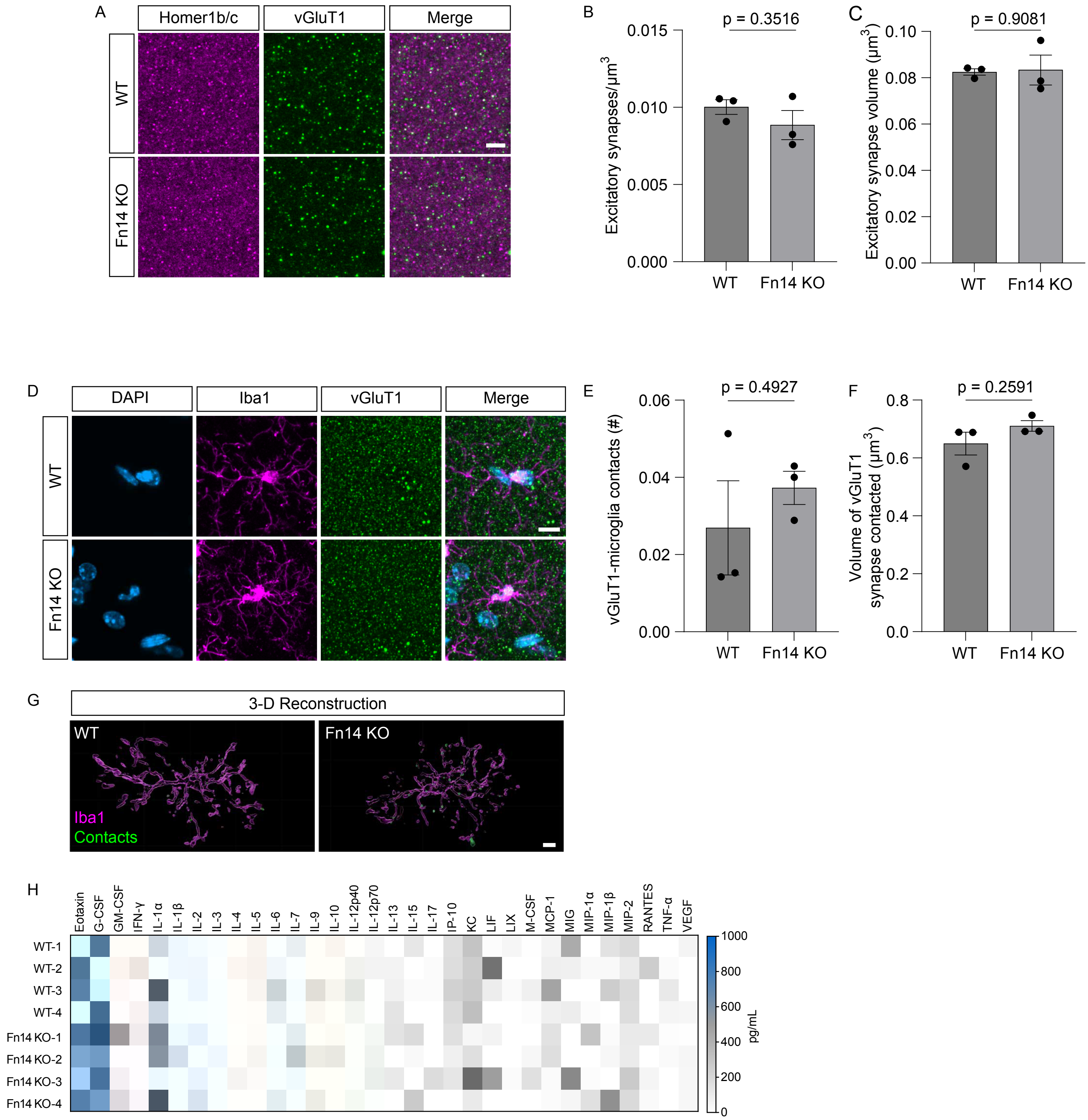

Figure S5 (Related to Figure 4). Fn14 KO does not affect excitatory synapses or microglia-synapse interactions in the developing hippocampus, or systemic cytokine expression in adult mice.

Figure S5 (Related to Figure 4). Fn14 KO does not affect excitatory synapses or microglia-synapse interactions in the developing hippocampus, or systemic cytokine expression in adult mice. (A) Confocal images of vGluT1 (green) and Homer1b/c (magenta) in WT and Fn14 KO mice between P21 and P24. Scale bar, 2 μm. (B),(C) Quantification of excitatory synapses per image volume (B) and the average volume of excitatory synapses (C). Unpaired Student's *t*-test, p-values on graphs; n = 3 mice/genotype. Data points are individual animals showing mean ± S.E.M. (D) Confocal images of microglia (Iba1, magenta), vGluT1 (green), and DAPI (blue) in WT and Fn14 KO mice between P21 and P24. Scale bar, 10 μm. (E),(F) Quantification of the number of vGluT1 synapse-microglia contacts (E) and average volumes of contacted synapses (F). Unpaired Student's *t*-test, p-values on graphs; n = 3 mice/genotype. Data points are individual animals showing mean ± S.E.M. Note that the highest datapoint in (E) is not a statistical outlier. (G) 3-D reconstructions of microglia (magenta) from images in (D) and vGluT1-microglia contacts (green). Scale bar, 10 μm. (H) Heat map depicting peripheral cytokine expression from adult Fn14 KO mice and WT littermate controls. Unpaired Student's *t*-test, $^*p < 0.05$; n = 4 mice/genotype.

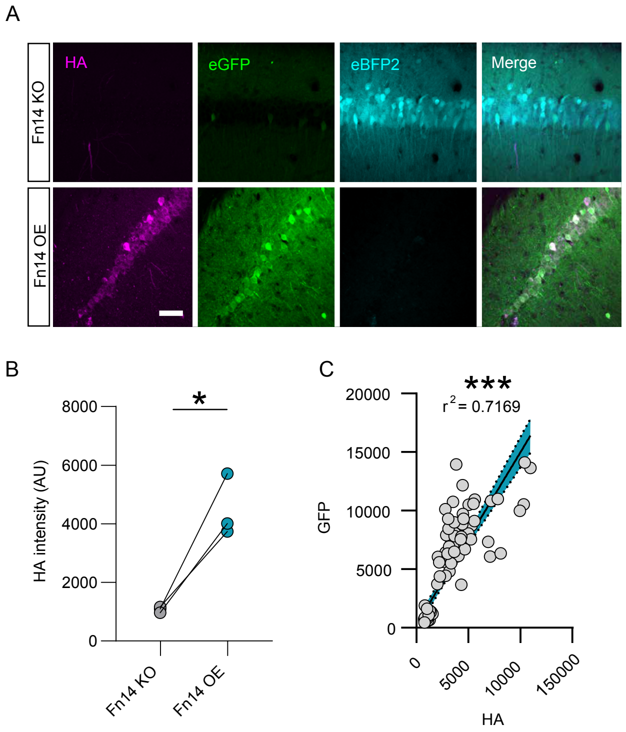

Figure S6 (Related to Figure 5). Additional validations of the Fn14 overexpression virus.

Figure S6 (Related to Figure 5). Additional validations of the Fn14 overexpression virus. (A) Example confocal images of CA1 in Fn14 KO mice injected with AAV9-hSyn-eBFP2 (control, top) and AAV9-hSyn-HA-Fn14-IRES-eGFP (bottom) into separate hemispheres. The HA tag is immunostained in magenta, eBFP2 is shown in blue, and eGFP is shown in green. Scale bar, 50 μm. (B) Quantification of HA fluorescence intensity in Fn14 KO (control) and Fn14 OE hemisphere. Paired *t*-test, *$p < 0.05$. Data points represent 3 individual hemispheres per condition from 3 mice. (C) Plot of eGFP by HA fluorescence intensities for each cell depicting a positive correlation. Pearson's correlation coefficients, $r^2 = 0.7169$, ***$p < 0.001$; $n = 117$ cells. Data points show individual cells with line of best fit and 95% confidence bands (teal region).

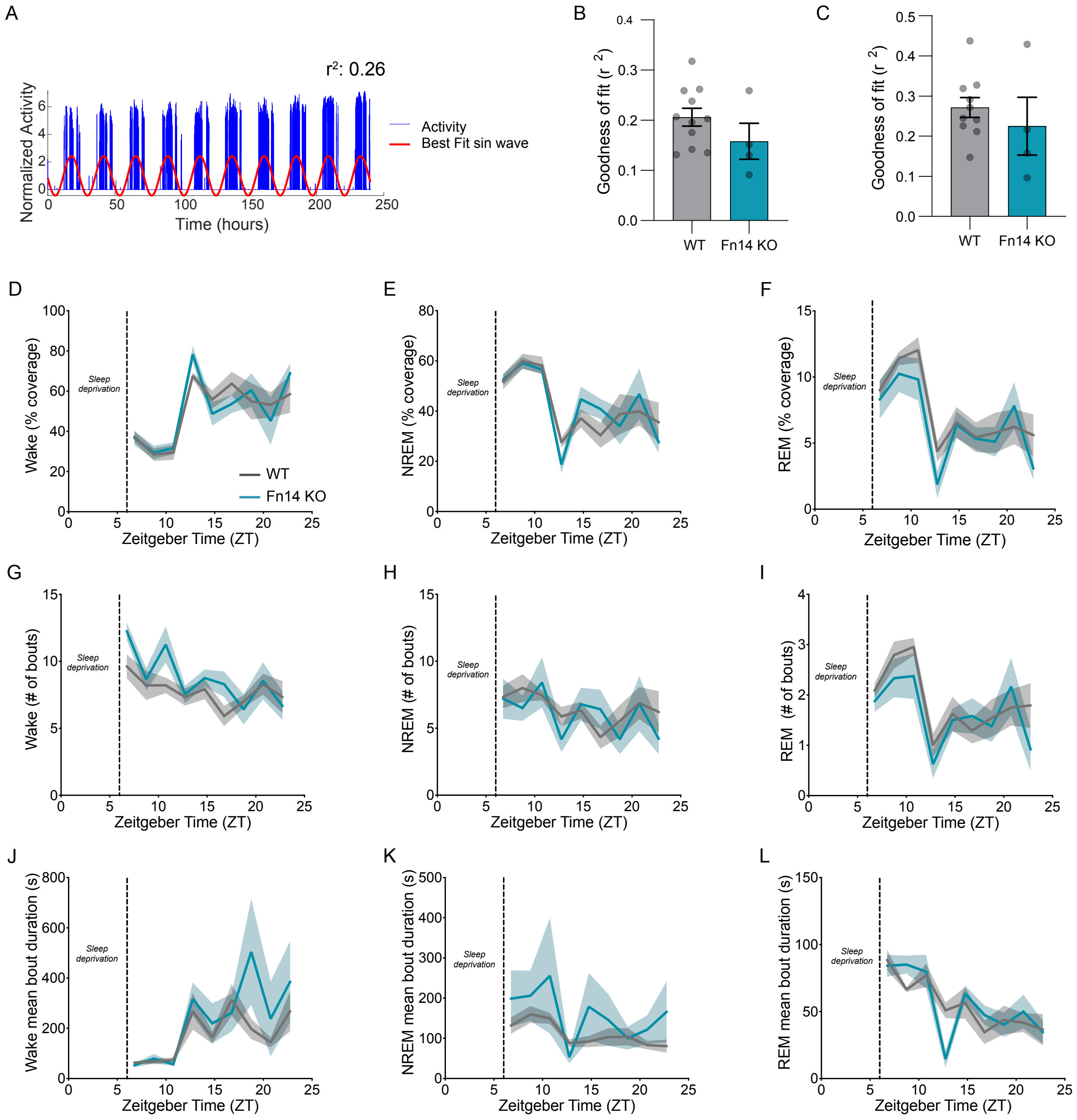

Figure S7 (Related to Figure 6). Goodness of fit of the sin wave in the analysis of running wheel data, and patterns of sleep recovery following sleep deprivation in Fn14 KO and WT mice.

Figure S7 (Related to Figure 6). Goodness of fit of the sin wave in the analysis of running wheel data, and patterns of sleep recovery following sleep deprivation in Fn14 KO and WT mice. (A) Example normalized activity data (blue) from an individual mouse over a 10-day period with the best fit sin wave (red). (B) Non-linear regression goodness of fit of WT and Fn14 KO mice in 12:12 light/dark. Welch's *t*-test, $p = 0.2883$. (C) Non-linear regression goodness of fit of WT and Fn14 KO mice in constant dark. Welch's *t*-test, $p = 0.5775$. (B),(C) Data points represent individual mice, mean ± S.E.M. is shown; n = 11 WT and 4 Fn14 KO mice. (D)-(F) Percent coverage of wake (D), NREM sleep (E), and REM sleep (F) in Fn14 KO and WT mice. (G)-(I) Number of wake (G), NREM sleep (H), and REM sleep (I) bouts in Fn14 KO and WT mice. (J)-(L) Bout duration for wake (J), NREM sleep (K), and REM sleep (L) in Fn14 KO and WT mice. (D)-(L) Line, mean; shaded area, S.E.M. Repeated measures two-way ANOVA with Šídák's multiple comparisons: time, genotype, and interaction, $p > 0.05$; n = 6 mice/genotype.
